# Phenotypic Differences between the Alzheimer’s Disease-Related hAPP-J20 Model and Heterozygous *Zbtb20* Knock-Out Mice

**DOI:** 10.1523/ENEURO.0089-21.2021

**Published:** 2021-05-10

**Authors:** Daniel R. Gulbranson, Kaitlyn Ho, Gui-Qiu Yu, Xinxing Yu, Melanie Das, Eric Shao, Daniel Kim, Weiping J. Zhang, Krishna Choudhary, Reuben Thomas, Lennart Mucke

**Affiliations:** 1 Gladstone Institute of Neurological Disease, Gladstone Institutes, San Francisco, CA 94158; 2 Department of Neurology and Weill Institute for Neurosciences, University of California, San Francisco, CA 94158; 3 Department of Pathophysiology, Naval Medical University, Shanghai 200433, China; 4 Gladstone Institute of Data Science and Biotechnology, Gladstone Institutes, San Francisco, CA 94158

**Keywords:** Alzheimer’s disease, amyloid precursor protein, behavior, epilepsy, mouse model, Zbtb20

## Abstract

Diverse gene products contribute to the pathogenesis of Alzheimer’s disease (AD). Experimental models have helped elucidate their mechanisms and impact on brain functions. Human amyloid precursor protein (hAPP) transgenic mice from line J20 (hAPP-J20 mice) are widely used to simulate key aspects of AD. However, they also carry an insertional mutation in noncoding sequence of one *Zbtb20* allele, a gene involved in neural development. We demonstrate that heterozygous hAPP-J20 mice have reduced Zbtb20 expression in some AD-relevant brain regions, but not others, and that Zbtb20 levels are higher in hAPP-J20 mice than heterozygous *Zbtb20* knock-out (*Zbtb20*^+/–^) mice. Whereas hAPP-J20 mice have premature mortality, severe deficits in learning and memory, other behavioral alterations, and prominent nonconvulsive epileptiform activity, *Zbtb20*^+/–^ mice do not. Thus, the insertional mutation in hAPP-J20 mice does not ablate the affected *Zbtb20* allele and is unlikely to account for the AD-like phenotype of this model.

## Significance Statement

Genetically modified mice can help unravel complex disorders such as Alzheimer’s disease (AD) by revealing effects of pathogenic drivers on neural networks and behaviors. Inadvertent genome modifications can occur during the generation of such models but their consequences are rarely explored in depth, although they could confound the interpretation of phenotypes and therapeutic interventions. Human amyloid precursor protein (hAPP) transgenic mice from line J20 (hAPP-J20 mice) simulate multiple aspects of AD but also carry an insertional mutation in one *Zbtb20* allele. Our study differentiates specific from nonspecific Zbtb20 antibodies and provides evidence that the functional AD-like alterations of hAPP-J20 mice are not caused by hypofunction of Zbtb20. We further demonstrate in *Zbtb20*^+/–^ mice that neural development and brain functions are well preserved when Zbtb20 levels are reduced in half.

## Introduction

Alzheimer’s disease (AD) is the most common neurodegenerative disorder and the most frequent cause of dementia (Brunnström et al., 2009; GBD 2016; Dementia Collaborators, 2019). In addition to the hardship AD inflicts on patients and caretakers, the disability and need for long-term care associated with this illness generate an enormous economic burden (Wimo et al., 2017; El-Hayek et al., 2019). Because the prevalence of AD is increasing around the world (GBD 2016; Dementia Collaborators, 2019), effective treatments are needed urgently. However, many investigational therapies have failed (Cummings et al., 2020; VandeVrede et al., 2020), most likely because of an incomplete understanding of AD pathogenesis and inadequate differentiation between clinicopathologic associations and true cause-and-effect relationships.

Animal models can help address these challenges. By revealing the *in vivo* effects and underlying mechanisms of suspected causal drivers of AD, they have complemented, expanded, and challenged conclusions drawn from clinical studies. For example, mouse models have shown that human amyloid precursor proteins (hAPPs), apolipoprotein E4, and tau can each cause neural network and cognitive dysfunctions independent of the formation of amyloid plaques and neurofibrillary tangles (Raber et al., 1998; Holcomb et al., 1999; Hsia et al., 1999; Mucke et al., 2000; Palop et al., 2003; Van Dam et al., 2003; Cleary et al., 2005; Cheng et al., 2007; Roberson et al., 2007; Wilcox et al., 2011; Huang and Mucke, 2012; Fowler et al., 2014; Maeda et al., 2016; Najm et al., 2019; Zott et al., 2019; Johnson et al., 2020; Chang et al., 2021). These findings may help explain why treatments aimed at plaques and tangles failed in clinical trials (Egan et al., 2019; Cummings et al., 2020; VandeVrede et al., 2020).

Heterozygous hAPP transgenic mice from line J20 (hAPP-J20 mice) are widely used to study the roles of hAPP and its metabolites in AD-related alterations of neural functions (Palop et al., 2003; Saganich et al., 2006; Ongali et al., 2010; Pozueta et al., 2013; Wright et al., 2013; Palop and Mucke, 2016; Flores et al., 2018; Ameen-Ali et al., 2019; Etter et al., 2019; Ferreira et al., 2020; Hanson et al., 2020; Johnson et al., 2020; Royea et al., 2020; Shabir et al., 2020). In this model, the platelet-derived growth factor (PDGF) β chain promoter directs neuronal expression of an alternatively spliced minigene encoding isoforms hAPP695, hAPP751, and hAPP770 each carrying mutations (“Swedish” and “Indiana”) that cause autosomal dominant familial AD (FAD) in humans (Rockenstein et al., 1995; Mucke et al., 2000). hAPP-J20 mice recapitulate many features of AD, including pathologically elevated levels of Aβ in AD-vulnerable brain regions, formation of amyloid plaques, neuritic dystrophy, gliosis, synaptic dysfunction and loss, nonconvulsive epileptiform activity, deficits in learning and memory, and other behavioral abnormalities (Mucke et al., 2000; Palop et al., 2003, 2007; Saganich et al., 2006; Cheng et al., 2007; Deipolyi et al., 2008; Ongali et al., 2010; Sanchez et al., 2012; Verret et al., 2012; Pozueta et al., 2013; Wright et al., 2013; Wilke et al., 2014; Orr et al., 2015; Palop and Mucke, 2016; Johnson et al., 2020).

**Figure 1.**
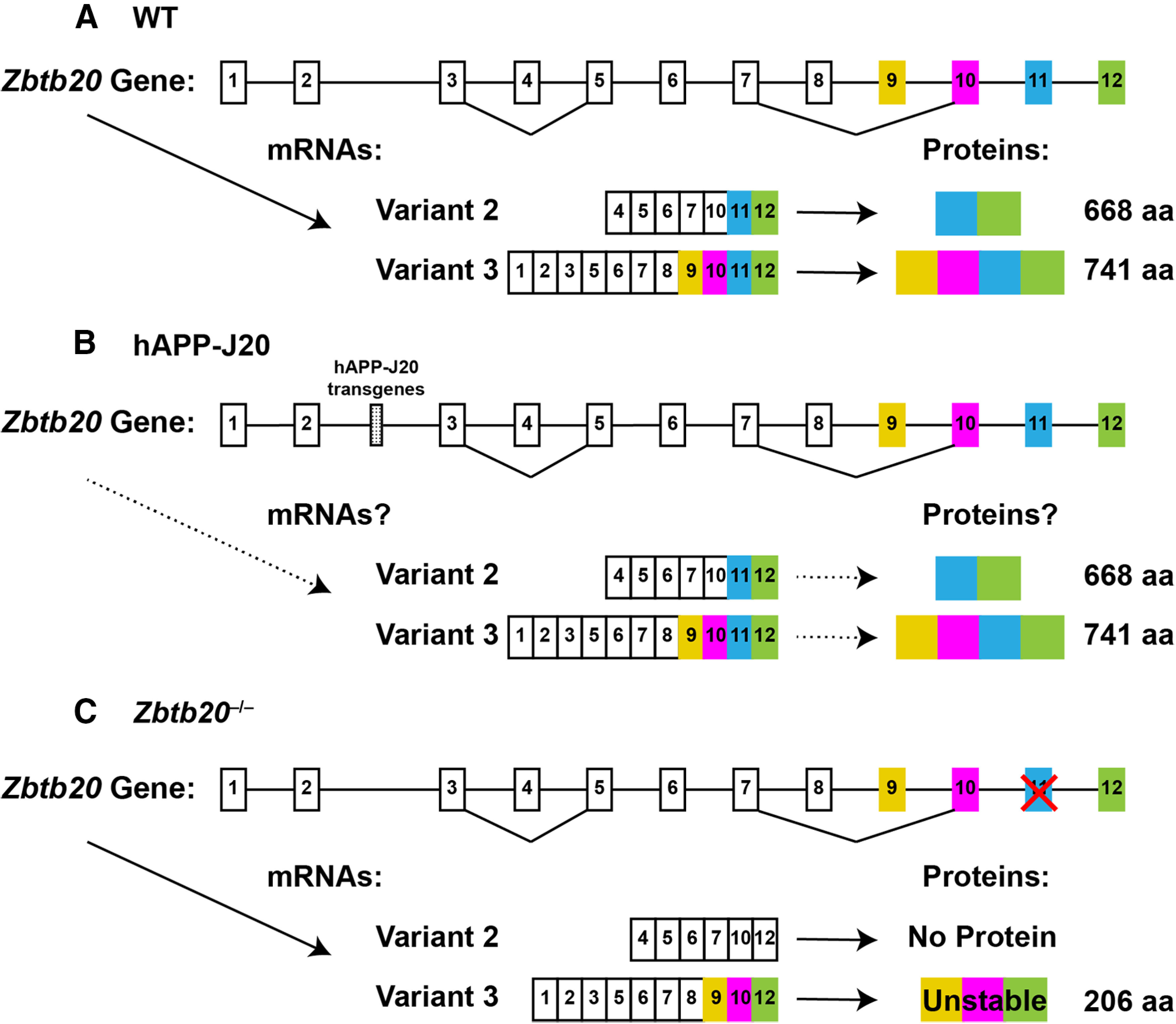
Simplified *Zbtb20* gene structure in WT, hAPP-J20, and *Zbtb20*^–/–^ mice. ***A–C***, Diagrammatic structure of the *Zbtb20* gene in WT mice (***A***), hAPP-J20 mice (***B***), and *Zbtb20*^–/–^ mice (***C***). Note that exons and introns are not drawn to scale and that putative translational start codons are present in exons 9 and 11, but not in exons 1–8 or 10. Translatable protein-coding exons are colored. ***A***, The WT *Zbtb20* gene (https://www.ncbi.nlm.nih.gov/gene/56490) has been shown to give rise to multiple alternatively spliced transcripts and two major protein products (668 and 741 amino acids (aa) in length, respectively) in cell lines (Mitchelmore et al., 2002). Examples of two confirmed *Zbtb20* mRNA variants and the proteins they encode are shown below. In principle, additional transcripts and protein isoforms might be derived from the *Zbtb20* gene in brain tissues (https://www.ncbi.nlm.nih.gov/gene/56490 and Wang et al., 2019). Probes and primers used to quantitate potentially protein-coding *Zbtb20* mRNAs by RT-qPCR were designed to detect sequences in exons 10 and 11 (Extended Data Fig. 1-1). ***B***, In hAPP-J20 mice, alternatively spliced minigenes (stippled box) that encode human hAPP695, hAPP751, and hAPP770 and are directed by the PDGF β chain promoter integrated into intron 2 of one endogenous *Zbtb20* allele. It is uncertain whether and how this insertional event affects the overall production of Zbtb20 mRNAs and proteins in heterozygous hAPP-J20 transgenic mice whose other *Zbtb20* allele is intact. ***C***, In *Zbtb20*^–/–^ mice, exon 11 was deleted, resulting in removal of ∼ 72% of the protein coding sequence (Sutherland et al., 2009).

However, most experimental models have flaws and limitations, and the hAPP-J20 model is probably no exception. A potential weakness of this line is its transgene integration into intron 2 of one allele of the zinc finger and BTB domain containing 20 (*Zbtb20*) gene (also known as *HOF*, *Znf288* and *Zfp288*), which caused an ∼41-kbp deletion of noncoding sequence (Fig. 1*A*,*B*; Tosh et al., 2018; Goodwin et al., 2019). Because *Zbtb20* is expressed in brain and the transcription regulator it encodes is involved in hippocampal development (Mitchelmore et al., 2002; Xie et al., 2010), it is conceivable that alterations in Zbtb20 expression could phenocopy AD-like functional alterations. We therefore studied the phenotype of heterozygous *Zbtb20* knock-out (*Zbtb20*^+/–^) mice (Fig. 1*C*; Sutherland et al., 2009; Xie et al., 2010) in relation to key abnormalities of hAPP-J20 mice, and compared the expression of Zbtb20 mRNAs and proteins in these models. We rigorously tested and refuted two main hypotheses: (1) the insertional mutation of hAPP-J20 mice knocks out the affected *Zbtb20* allele and lowers Zbtb20 levels in AD-relevant brain regions to those found in *Zbtb20*^+/–^ mice, and (2) *Zbtb20*^+/–^ mice develop prominent neural network and behavioral abnormalities similar to those observed in hAPP-J20 mice. Our findings support the conclusion that AD-like functional alterations in hAPP-J20 mice are not caused by *Zbtb20* hypofunction and, instead, suggest that these alterations are mostly caused by the transgene products expressed in this model.

10.1523/ENEURO.0089-21.2021.f1-1 Extended Data Figure 1-1 Key reagents. Download Figure 1-1, DOCX file.

## Materials and Methods

### Mice

The generation of hAPP-J20 mice (Mucke et al., 2000) and *Zbtb20*^+/–^ mice (Sutherland et al., 2009) was described previously. 5XFAD mice (Oakley et al., 2006) and APP/PS1 mice (Jankowsky et al., 2004) were obtained from The Jackson Laboratory (008730 and 005864, respectively). All mice were maintained on a C57BL/6J background by breeding heterozygous transgenic or heterozygous knock-out mice with wild-type (WT) C57BL/6J mice from The Jackson Laboratory (000664). Controls that were WT for *Zbtb20* and lacked the hAPP-J20 transgene were generated by breeding heterozygous male hAPP-J20 mice with WT female C57BL/6J mice, or by breeding male *Zbtb20*^+/–^ mice with female *Zbtb20*^+/–^ mice. Groups of genetically modified mice and WT controls were always generated by the breeding schemes described above, although not all genetically modified mice and WT controls compared in individual experiments were raised by the same dam at the same time (littermates). For each experiment and line, experimental and control groups included roughly comparable proportions of males and females, unless indicated otherwise in figure legends. Mice were fed a regular chow diet (PicoLab Rodent Diet 5053, TestDiet) and maintained on a 12/12 h light/dark cycle. Mice were weaned three to four weeks after birth and housed in cages containing up to five mice. The survival of mice was monitored from the time they reached one month of age until they were killed. All animal experiments were approved by the Institutional Animal Care and Use Committee of the University of California, San Francisco.

### Tissue processing

Mice were anesthetized with 125–400 mg/kg 2,2,2-tribromoethanol (Avertin; Alfa Aeser, A18706) and perfused with 0.9% saline. Livers were snap frozen on dry ice and hemibrains were either snap frozen or drop-fixed in 4% paraformaldehyde (Electron Microscopy Sciences, 15714)/PBS for 48 h at 4° C. Fixed hemibrains were transferred to 30% sucrose/PBS for 1–2 d at 4° C and 30-μm sections were obtained with a freezing microtome (Leica, SM2000R). Sections were stored in a cryoprotectant solution consisting of 30% ethylene glycol (Fisher Scientific, E178-4), 30% glycerol (Fisher Scientific, G33-4) and 40% PBS (Corning, 46-031-CM) at –20° C until further use. Frozen hemibrains were thawed for 1 h on ice before dissection of the hippocampus and cortex, which were used for Western blotting or RT-qPCR.

### Western blotting

Tissues were homogenized with a Storm 24 bullet blender tissue homogenizer (Next Advance) in Pierce RIPA buffer (Thermo Fisher Scientific, 89901) with protease inhibitor (Sigma, 11836153001) and phosphatase inhibitor cocktails 2 & 3 (Sigma-Aldrich, P5726, P0044) and sonicated with an EpiSonic multifunctional bioprocessor (EpGentek) for 10 min at an amplitude of 40 (∼140 W). Lysates were centrifuged at 10,500 × *g* for 10 min, supernatants collected, and protein concentrations measured by Bradford assay (Bio-Rad, 5000006). A total of 20 μg of protein was diluted in 20 μl of NuPAGE sample buffer (Invitrogen, NP0007) containing NuPAGE sample reducing agent (Invitrogen, NP0009), heated to 70° C for 10 min, and loaded on NuPAGE 4–12% Bis-tris gels (Invitrogen, WG1403BX10). Samples were electrophoresed at 180 V for ∼1 h in a XCell4 SureLock Midi-Cell gel electrophoresis system (Invitrogen, WR0100) filled with 1× NuPAGE MOPS SDS running buffer (Invitrogen, NP0001-02). After electrophoresis, proteins were transferred to a nitrocellulose membrane (Bio-Rad, 1620112) at 4° C for ∼12 h at 0.15 A using a Criterion Blotter (Bio-Rad, 1704070) or for 10 min at 25 V with an iBlot 2 (Thermo Fisher Scientific, IB21001). Membranes were blocked with 5% non-fat dry milk in tris-buffered saline (TBS) for 1 h at room temperature and incubated overnight at 4° C in 5% non-fat dry milk/TBS containing 0.1% Tween 20 and primary antibodies as indicated in Extended Data Figure 1-1. The next day, blots were washed 4×, alternating between TBS and TBS with 0.1% Tween 20, and incubated at room temperature for 1 h in TBS containing 0.2% Tween 20, 50% Odyssey blocking buffer (LI-COR, 927-5000) and secondary antibodies as indicated in Extended Data Figure 1-1. After washing 4× as before, blots were scanned with an Odyssey CLx Imager (LI-COR). Signal intensities were quantified with Image Studio version 5.2.5 software (LI-COR).

### RT-qPCR

Total RNA was isolated from tissues with RNeasy kits (QIAGEN, 74 106) and reverse-transcribed with TaqMan Reverse Transcription Reagents (Thermo Fisher Scientific, N8080234). Quantitative real-time PCR was performed on an ABI Prism 7900HT Sequence Detection System (Thermo Fisher Scientific) using Power SYBR Green Nucleic Acid Detection kits (Thermo Fisher Scientific, 4367659) or TaqMan Gene Expression Master Mix (Thermo Fisher Scientific, 4368706) with RT-qPCR primers or TaqMan probes indicated in Extended Data Figure 1-1. Relative target mRNA levels were calculated with the 2^–ΔΔC^_T_ method (Livak and Schmittgen, 2001) using *Gapdh* mRNA as the internal reference and graphed relative to mean values in the control group.

### snRNA-seq

Mice were anesthetized and perfused and the hippocampus and cortex from one hemibrain were immediately dissected without freezing. Fresh samples were incubated on ice for 10 min in a solution containing 0.25 M sucrose (Sigma, S0389), 25 mM KCl (Sigma, P9333), 5 mM MgCl_2_ (Sigma, M8266), 20 mM Tricine-KOH at pH 7.8 (Sigma, T9784 and P1767), 0.5% Triton X-100 (Sigma-Aldrich, X100), 1 mM DTT (Sigma, 11583786001), 800 units RNase Inhibitor (Lucigen, 30 281–2), and 1× protease inhibitor (Sigma, 11836153001), homogenized with a Wheaton Dounce Tissue Grinder (Fisher, 06-435A) using the supplied loose pestle 5× and tight pestle 12×; filtered through a 40-μm pore-size filter, and centrifuged at 500 × *g* for 10 min. Samples were resuspended in a solution containing the same components listed above plus 30% iodixanol (Sigma, D1556), centrifuged at 10,000 × *g* for 20 min at 4° C, and resuspended in PBS containing 1% BSA (Sigma, A8806) and 0.2 U/μl RNase Inhibitor at 1000 nuclei/μl. mRNA from individual nuclei was reverse transcribed, and barcodes and Illumina adaptors were added to the resulting cDNA in an emulsion by loading 15 μl of nuclei suspension on a Chromium Controller (10× Genomics) and following the manufacturer’s protocol for the Chromium Next GEM Single Cell 3′ kit v3.1(10× Genomics, PN-1000121). Sequencing libraries were sequenced on a NextSeq 500 (Illumina) following the manufacturer’s protocol for the NextSeq 500/550 Mid Output V2.5 kit (150 cycles; Illumina, 20024904) to assess the distribution of reads across samples. After re-balancing, library pools were sequenced on a NovaSeq 6000 (Illumina) using a NovaSeq 6000 S4 Reagent kit v1.5 (200 cycles; Illumina, 20028313) resulting in an average read depth of 20,000 reads per nucleus.

Reads from each sample were aligned to the mm10 mouse reference (version 2020-A from 10× Genomics website) using Cell Ranger version 3.1.0. The resulting count matrices for all samples were aggregated without depth normalization. The aggregated matrix was analyzed with the Seurat package (version 3.1.3.9002) in R (Stuart et al., 2019). The Seurat object was created after removing features detected in fewer than three nuclei as well as nuclei with fewer than 200 detected features. After assessing the distribution of quality metrics, nuclei with >2000 features and >5% mitochondrial counts were removed. The remaining counts were normalized with the LogNormalize method using scale-factor 10,000, and 2000 highly variable features were selected with the “vst” method. The normalized counts were scaled and centered, and principal component analysis was performed for the selected features. Clustering was done using a shared nearest neighbor graph built with the top 20 principal components and the original Louvain algorithm for modularity optimization with resolution parameter set to 1.2. UMAP embedding was then generated using the top 20 principal components. If the median number of nuclei per mouse in a cluster was <20, the cluster was not shown.

### Immunohistochemistry

For immunofluorescence experiments, sections were rinsed 2× in PBS to remove cryoprotectant, blocked at room temperature for 1 h with 5% normal goat serum (Jackson ImmunoResearch, 005-000-121) in PBS, and incubated overnight with gentle shaking at 4° C in PBS containing 3% normal goat serum and primary antibodies as indicated in Extended Data Figure 1-1. The next day, sections were washed 4×, alternating between PBS and PBS with 0.25% Triton X-100, incubated for 1 h at room temperature in PBS containing 3% normal goat serum and secondary antibodies as indicated in Extended Data Figure 1-1, and washed 4× as before. Sections were mounted on slides and coverslipped with Prolong Diamond mountant (Thermo Fisher Scientific, P36970). The above protocol was applied to all immunofluorescent antibodies except for anti-Iba1, which required an additional signal amplification step. For this antibody, we used a TSA Plus Cyanine 5 detection kit (Akoya Biosciences, NEL745001KT) following the manufacturer’s protocol. Images were captured with an Olympus FV3000 Laser Scanning confocal microscope (Olympus), a BZ-X710 All-in-One Fluorescence microscope (Keyence), or an Aperio VERSA slide scanner (Leica Biosystems). Signal intensity was quantified with ImageJ software (https://imagej.net/Fiji). Signal intensities were averaged from three sections per mouse.

Immunohistochemistry experiments with colorimetric readouts followed established protocols (Palop et al., 2011). Sections were rinsed 2× in PBS to remove cryoprotectant, quenched with PBS containing 3% hydrogen peroxide and 10% methanol for 15 min at room temperature, and blocked for 1 h in PBS containing 1% non-fat dry milk, 0.2% gelatin, 0.5% Triton X-100, and 10% normal donkey serum. For c-Fos immunostaining, sections were incubated in citrate buffer (9.4 mM citric acid, 41 mM sodium citrate, pH 6) for 10 min at 100° C before the blocking step for antigen retrieval. All sections were incubated overnight at 4° C in PBS containing 0.2% gelatin, 0.5% Triton X-100, 3% normal donkey serum, and primary antibodies as indicated in Extended Data Figure 1-1. The next day, sections were washed 4×, alternating between PBS and PBS with 0.25% Triton X-100, and incubated for 1 h at room temperature in PBS containing 0.2% gelatin, 0.5% Triton X-100, 3% normal donkey serum, and biotinylated anti-rabbit or anti-mouse secondary antibodies as indicated in Extended Data Figure 1-1. After washing 4× as above, signal was enhanced and visualized with Elite ABC (Vector Laboratories, PK6100) and 3,3′-diaminobenzidine (DAB) tetrahydrochloride kits (Vector Laboratories, SK4100). Bright-field images were captured on an Aperio VERSA slide scanner (Leica Biosystems) with a 10× objective. c-Fos positive cells were counted by an investigator and optical density measurements of calbindin and neuropeptide Y (NPY) immunoreactivity in dentate gyrus were quantified using ImageJ software version 1.47 (http://imagej.nih.gov/ij).

### Behavioral testing

#### Morris water maze

Mice were given four consecutive pretraining trials during which they swam down a rectangular track (15 × 122 cm) and mounted a hidden platform (14 × 14 cm) submerged 1.5 cm below the water surface. Mice that did not find the platform were guided to the platform and allowed to sit on it for 10 s. After pretraining, mice were trained for 5 d to locate a stationary hidden platform in a circular pool (122 cm in diameter) filled with water (21 ± 1° C) that was opacified with a non-toxic white tempera paint. Distinct extra-maze cues surrounded the pool. Each day, mice were trained in two sessions comprising two trials. Sessions were separated by 3 h, and the intertrial interval was 15 min. Mice were placed in the pool at predetermined drop locations that varied among trials. Trials ended when mice mounted the hidden platform or 60 s had passed. Mice that did not find the platform were guided to it by the investigator. All mice were allowed to sit on the hidden platform for 10 s after each trial.

Probe trials were conducted 24 and 72 h after the final day of hidden platform training. For each probe trial, the hidden platform was removed and the mice were placed into the pool opposite the former platform location. After 60 s, the investigator retrieved the mice from where the hidden platform was located during the training phase. After the probe trials, mice were trained for 2 d to locate a visibly cued platform (15-cm striped pole on top of a platform) whose position was changed between sessions. Each day, mice were trained in three sessions comprising two trials. Sessions were separated by 3 h and the intertrial interval was 15 min. EthoVision XT video tracking (Noldus Information Technology) was used to analyze swim paths, escape latencies, and swim speeds.

#### Active place avoidance

The testing chamber consisted of a clear acrylic enclosure (40 cm in diameter) positioned in the center of a square rotating arena (81 × 81 cm; Bio-Signal Group Corp.) with a grid floor and was surrounded by black-and-white spatial cues. A 60° sector within the acrylic enclosure was fixed relative to the spatial cues and designated as the aversive zone. Training occurred over four consecutive days with one 10-min trial per day. During each trial, mice were placed in the enclosure, which rotated at 1 rpm, and their position was monitored by video using Tracker (Bio-Signal Group Corp.). The first training day was considered a habituation trial and no foot shock was administered if the mice entered the aversive zone. On days 2–4, if the mice entered the aversive zone, a 0.2-mA shock was delivered for 500 ms every 1.5 s until the mice left the aversive zone; 24 h after the final training day a 5-min probe trial was conducted in which the current source was turned off. The chamber was cleaned with 70% ethanol between mice.

#### Elevated plus maze

Mice were transferred to the dimly-lit testing room and allowed to acclimate for 1 h. The maze (Kinder Scientific) was positioned 63 cm above the ground and consisted of four 38-cm-long arms that intersected at 90°. Two arms were open (without walls) and two arms were closed (with side walls). After acclimation, mice were placed at the intersection of the arms and allowed to explore the maze for 10 min. Distance traveled and time spent in open and closed arms were monitored with infrared photobeams.

#### Open field

Activity in a clear acrylic chamber (41 × 41 × 30 cm) was measured with an Open Field Photobeam Activity System (San Diego Instruments), which consisted of a 16 × 16 photobeam array to automatically monitor movements. After allowing the mice to acclimate to the testing room, they were placed in the center of the chamber and allowed to explore for 15 min. Movements, including locomotion and rearing, were recorded automatically by the photobeam array.

### EEG recording and analysis

EEG electrodes were produced in-house by soldering Teflon-coated silver wire electrodes (0.125 mm in diameter) to a four-channel electrical connector. After mice were anesthetized with isoflurane, EEG electrodes were surgically implanted beneath the skull over the parietal and frontal cortices. Stereotaxic coordinates relative to the bregma were –2 mm anteroposterior (AP) and ±2 mm mediolateral (ML) and +1 mm AP and ±1 mm ML, respectively. Before EEG recordings were made, mice were allowed to recover from the surgery in their home cages for at least two weeks. Electrical signals between the left parietal cortex and left frontal cortex (reference electrode) were amplified with a Differential Amplifier (Warner Instruments) and recorded with a PowerLab data acquisition system (AD Instruments), which also integrated video recordings of the mice. All signals were acquired at a sampling rate of 1000 Hz. EEG and video recorded for ∼8 h in resting mice during the light cycle were analyzed for epileptiform spikes with a macro written in LabChart 8 Pro software (AD Instruments). EEG signals were filtered with a 5- to 50-Hz bandpass filter to eliminate any electrical noise. Spikes were identified by their sharpness and amplitude relative to baseline signal; the latter was defined as the average of the square-root of the signal within 20 s. If the square-root of a change in amplitude during 0.02 s was more than ±4× baseline and the absolute value of the second derivative of the slope was ±10^4^, a potential epileptiform spike was indicated. An investigator reviewed videos and EEG signals to exclude spike-like deflections that likely resulted from movement-related artifacts. Spike frequency was calculated from the total number of verified spikes detected during the total time mice spent resting in the ∼8-h recording period.

### Liver function indicators

Mice were anesthetized with Avertin and ∼500 μl of blood was taken from the right atrium of the heart, mixed in a BD Microtainer tube containing lithium heparin (BD, 365958), and centrifuged at 500 × *g* for 7 min. Serum was collected and stored at –70° C until further use. Serum chemistry profiles were determined by IDEXX BioAnalytics using an AU680 Chemistry System (Beckman Coulter). Serum levels of alanine aminotransferase (ALT) activity, aspartate aminotransferase (AST) activity, total bilirubin, and albumin (ALB) were determined enzymatically using reagents designed for the AU680 Chemistry System and following the manufacturer’s instructions.

### Experimental design and statistical analysis

Investigators were blinded to the genotype of mice. For Western blotting and RT-qPCR measurements, samples were distributed semi-randomly across lanes and wells, respectively, to avoid clustering samples of the same genotype. Group sizes for *Zbtb20* mRNA measurements were determined based on a pilot experiment in which hippocampal and cortical *Zbtb20* mRNA levels were compared in WT and hAPP-J20 mice (*n *=* *7–11 per group) and found to be reduced by ∼35% in the hippocampus and ∼10% in the cortex of hAPP-J20 mice (data not shown). Based on these reductions and the variances observed, a power analysis using a significance level of 0.05 and a power of 0.8 indicated that using nine mice per group would provide the power to detect a 15% difference in *Zbtb20* mRNA levels in either brain region. For measurements of doublecortin and liver function indicators, group sizes were based on available samples. Group sizes for behavioral assays, survival curves, EEG recordings, and molecular indicators of epileptiform activity were all based on findings previously reported for hAPP-J20 mice. Group sizes in the snRNA-seq experiment were suboptimal because of budget constraints.

Statistical tests were performed with Prism 8 or 9 (GraphPad Software) or the statistical programming language R (http://www.R-project.org/) and are indicated in the figure legends. Data distribution was assessed with the D’Agostino–Pearson test. Parametric tests were used on normally distributed data. If data were not normally distributed, normality was evaluated on log transformed data. If the transformation resulted in a normal distribution, parametric tests were used on the transformed data. If the transformed data were not normally distributed, non-parametric tests were used on the untransformed data. Variance among groups was assessed with Bartlett’s test (normal distribution) or Brown–Forsythe test (non-normal distribution). Normally distributed data with equal variance was analyzed by paired or unpaired two-tailed Student’s *t* test, normally distributed data with unequal variance by unpaired two-tailed *t* test with Welch correction, and non-normally distributed data by two-tailed permutation test. For group sizes <10, parametric tests were used because we could not reliably assess data distribution. Data comprising repeated measures were assessed by Mantel–Cox log-rank test (survival curves) or linear mixed model analysis with Holm–Sidak correction for the comparison of specific groups. For snRNA-seq data, the significance of genotype effects on *Zbtb20* expression was determined in clusters whose medians of *Zbtb20* expression were >0 in ≥3 WT mice using the R package *muscat* (version 1.0.1; Crowell et al., 2020). For each cluster, raw read counts from all nuclei of any given mouse were combined to obtain pseudo-bulk RNA-seq counts. Unadjusted *p* values for the differential expression of *Zbtb20* between mice of different genotypes were extracted from the *muscat* results for each cluster and a Holm–Sidak correction was performed to correct for multiple comparisons, using individual mice as the number of biological samples (*n*).

## Results

### Zbtb20 expression in mouse brain and liver

**Figure 2.**
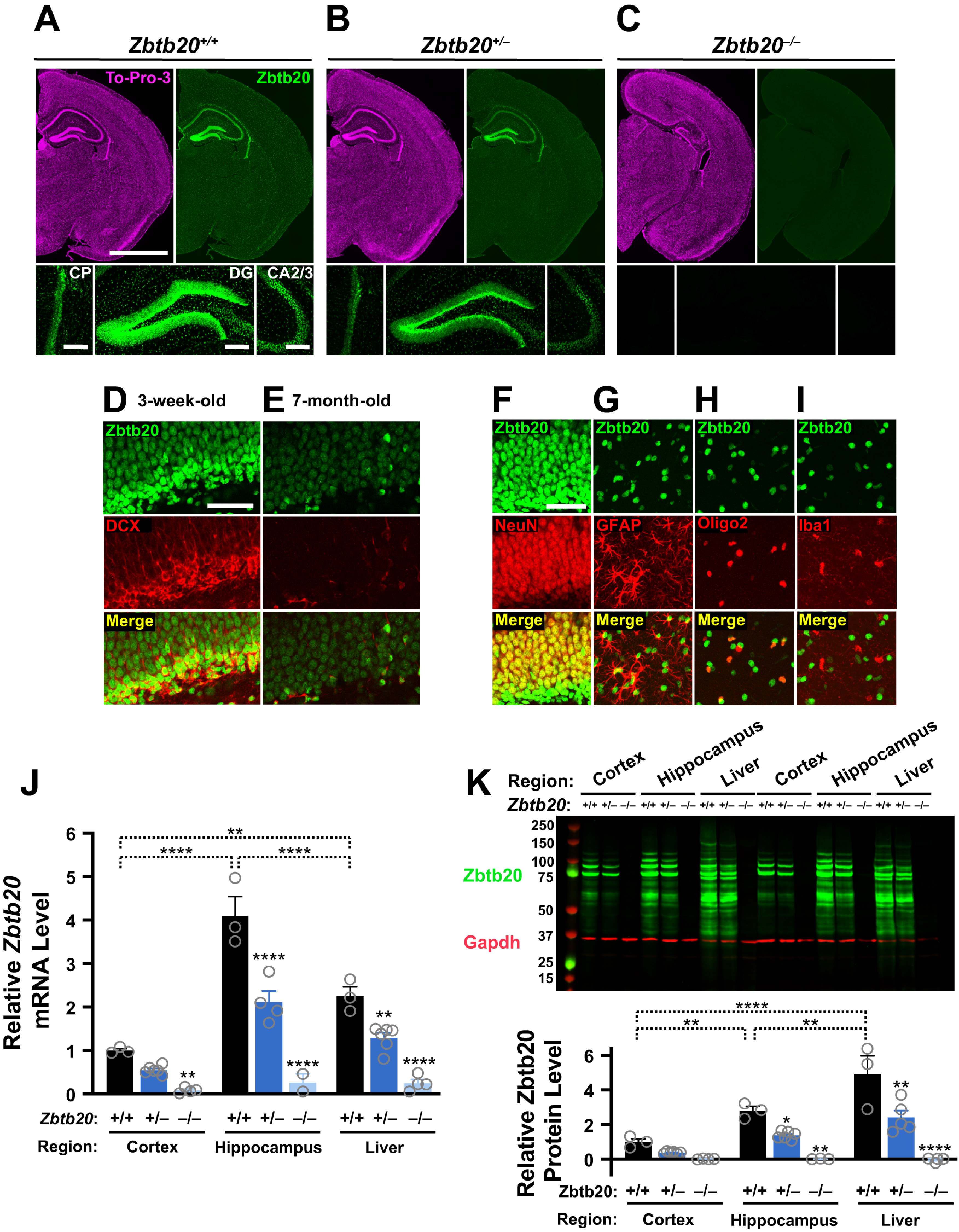
Zbtb20 mRNA and protein expression in *Zbtb20*^+/+^, *Zbtb20*^+/–^, and *Zbtb20*^–/–^ mice. ***A–C***, Coronal brain sections from three-week-old mice of the indicated genotypes were stained with TO-PRO-3 to identify nuclei (top-left in each panel), labeled with an antibody (BD Biosciences, catalog #565453) against Zbtb20 (top-right and bottom row in each panel), and imaged by epifluorescence microscopy. Hemibrain sections (scale bar: 2 mm) are shown at the top and magnified views (scale bar: 200 μm) of the choroid plexus (CP; left), dentate gyrus (DG; center), and CA2/3 (right) at the bottom. ***D***,***E***, Coronal brain sections from three-week-old (***D***) and seven-month-old (***E***) WT mice were co-immunostained for Zbtb20 and doublecortin (DCX) and imaged by confocal microscopy. The subgranular zone of the dentate gyrus is shown (scale bar: 50 μm). ***F–I***, Coronal brain sections from three-week-old WT mice were immunostained for Zbtb20 and co-labeled with antibodies against cell type-specific markers for (***F***) neurons (NeuN), (***G***) astrocytes (GFAP), (***H***) oligodendrocytes (Oligo2), or (***I***) microglia/macrophages (Iba1), and imaged by confocal microscopy. The granular layer (***F***) and molecular layer (***G–I***) of the dentate gyrus are shown (scale bar: 50 μm). ***J***,***K***, *Zbtb20* mRNA (***J***) and Zbtb20 protein (***K***) levels in cortices, hippocampi and livers from three-week-old mice of the indicated genotypes were determined by RT-qPCR and Western blotting, respectively. ***J***, Relative *Zbtb20* mRNA levels were quantified by the 2^–ΔΔC^_T_ method (Livak and Schmittgen, 2001) using *Gapdh* mRNA as the internal reference. Mean *Zbtb20/Gapdh* mRNA ratios in WT cortical samples were defined as 1.0; *n* = 2–6 mice per group. ***K*,** Western blot depicting Zbtb20 signals across tissues and genotypes is shown on top. Samples from replicate groups of mice were loaded on the left and right part of the gel, respectively. Gapdh was used as a loading control. Quantitations of Western blot signals are shown below. Zbtb20 signals were normalized to Gapdh signals and mean Zbtb20/Gapdh ratios in the cortex of WT mice were defined as 1.0; *p <* *0.05, **p <* *0.01, ****p <* *0.0001 versus WT mice or as indicated by brackets (linear mixed model analysis with Holm–Sidak correction). Dots represent individual mice and bars are means ± SEM.

**Figure 3.**
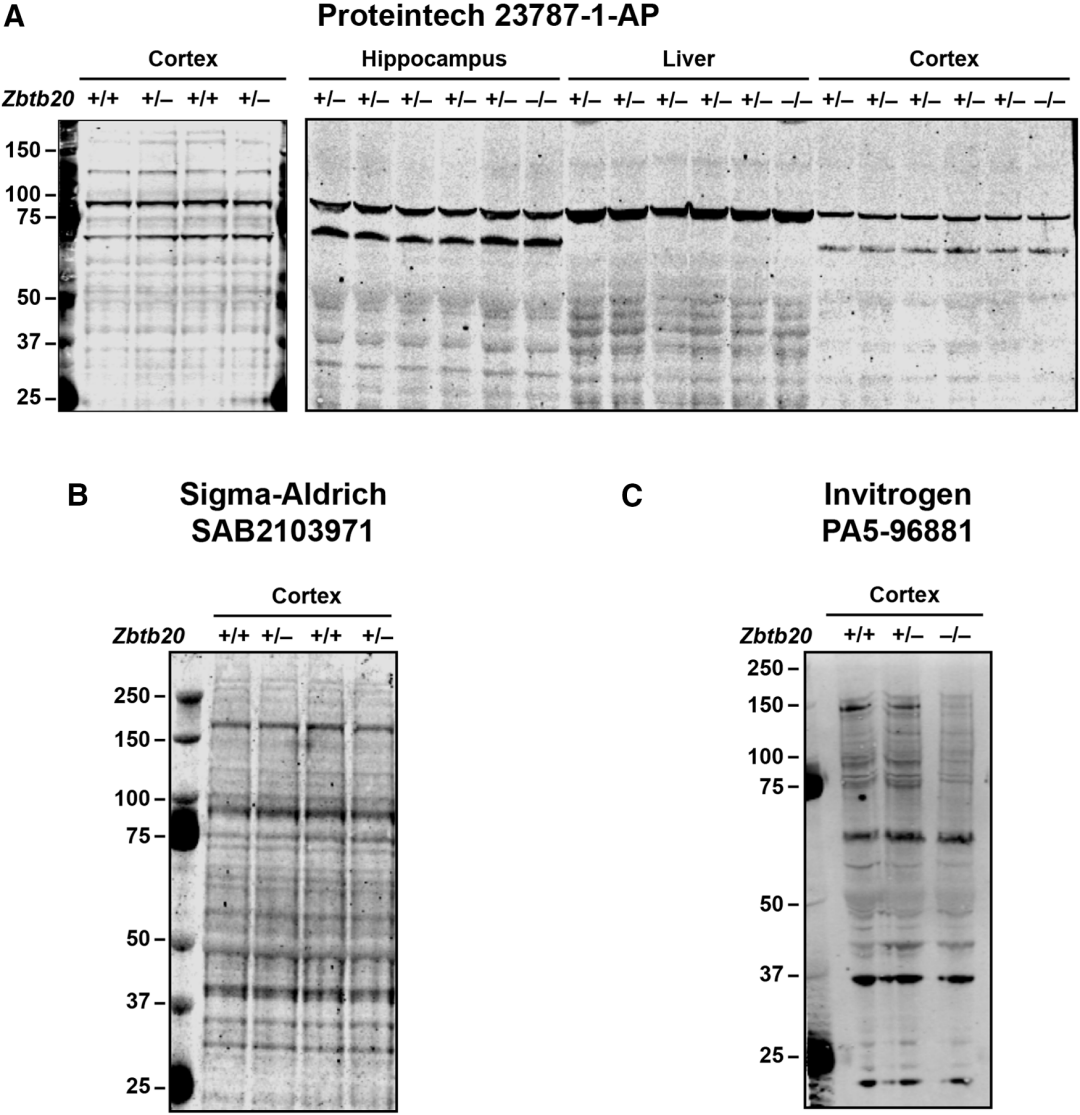
Several other antibodies raised against Zbtb20 lack specificity. ***A–C***, Western blots depict representative immunoreactivity patterns detected with the anti-Zbtb20 antibodies listed above the respective blots in lysates of cortex (***A***–***C***), hippocampus (***A***), and liver (***A***) from three-week-old mice of the indicated genotypes. Note that genetic modulation of Zbtb20 expression did not alter the staining obtained with these antibodies.

To assess the specificity of anti-Zbtb20 antibodies, we tested them on brain and liver tissues from *Zbtb20*^+/+^, *Zbtb20*^+/–^, and *Zbtb20*^–/–^ mice. Because a Zbtb20 antibody from BD Biosciences (catalog #565453) showed excellent specificity by immunostaining of tissue sections and Western blot analysis of tissue lysates (Fig. 2), this reagent was chosen for the detection and quantification of Zbtb20 in subsequent experiments. In contrast, several other antibodies raised against Zbtb20 lacked specificity (Fig. 3), including antibody #23787-AP from ProteinTech, which was previously used to compare hippocampal Zbtb20 levels in non-transgenic (NTG) and hAPP-J20 mice (Tosh et al., 2018).

In brains of WT *Zbtb20*^+/+^ mice, Zbtb20 was expressed predominantly in the hippocampus, consistent with previous reports (Nielsen et al., 2010; Xie et al., 2010), and choroid plexus (Fig. 2*A*). Zbtb20 expression in these structures was gene dose-dependently reduced in *Zbtb20*^+/–^and *Zbtb20*^–/–^ mice (Fig. 2*B*,*C*). *Zbtb20*^–/–^ mice had an abnormal hippocampal cytoarchitecture (Fig. 2*C*), consistent with previous findings (Xie et al., 2010), but this alteration was not present in *Zbtb20*^+/–^mice (Fig. 2*B*). In the hippocampus of neonatal WT mice, Zbtb20 expression was most prominent in the granular layer of the dentate gyrus, particularly in granule cells co-expressing doublecortin (Fig. 2*D*), a marker of newborn neurons (Couillard-Despres et al., 2005). Adult WT mice had lower levels of neuronal Zbtb20 expression and fewer doublecortin-positive granule cells (Fig. 2*E*). Although Zbtb20 expression was most prominent in neurons (Fig. 2*F*), it was also detectable in astrocytes (Fig. 2G) and oligodendrocytes (Fig. 2*H*), but not in microglia (Fig. 2*I*), as demonstrated here in three-week-old mice.

*Zbtb20* mRNA levels in WT mice were higher in hippocampus than cortex and liver (Fig. 2*J*). As expected, *Zbtb20* mRNA levels were ∼50% lower in *Zbtb20*^+/–^micethan in *Zbtb20*^+/+^ controls and at background levels in *Zbtb20*^–/–^ mice (Fig. 2*J*). Using the same antibody that yielded reliable Zbtb20 immunostaining of brain sections (Fig. 2*A–I*), we detected multiple immunoreactive bands by Western blot analysis in lysates of cortex, hippocampus and liver from *Zbtb20*^+/+^ and *Zbtb20*^+/–^ mice, but not *Zbtb20*^–/–^ mice. The most prominent bands in cortex and hippocampus were between 75 and 100 kDa (Fig. 2*K*). Additional Zbtb20 bands above 100 kDa and below 75 kDa (Fig. 2*K*) may represent different Zbtb20 isoforms, posttranslational modifications, or metabolites. Because all bands were gene dose-dependently reduced in *Zbtb20*^+/–^ and *Zbtb20*^–/–^ mice (Fig. 2*K*), they likely represent genuine Zbtb20 species rather than nonspecific cross reactivities. Since no Zbtb20-immunoreactive signals were detected in *Zbtb20*^–/–^ mice (Fig. 2*K*), the small protein product that might be expressed from the modified *Zbtb20* gene in *Zbtb20*^–/–^ mice (Fig. 1*C*) is either not made or too unstable for detection.

### Reduced hippocampal Zbtb20 expression in hAPP-J20 mice

**Figure 4.**
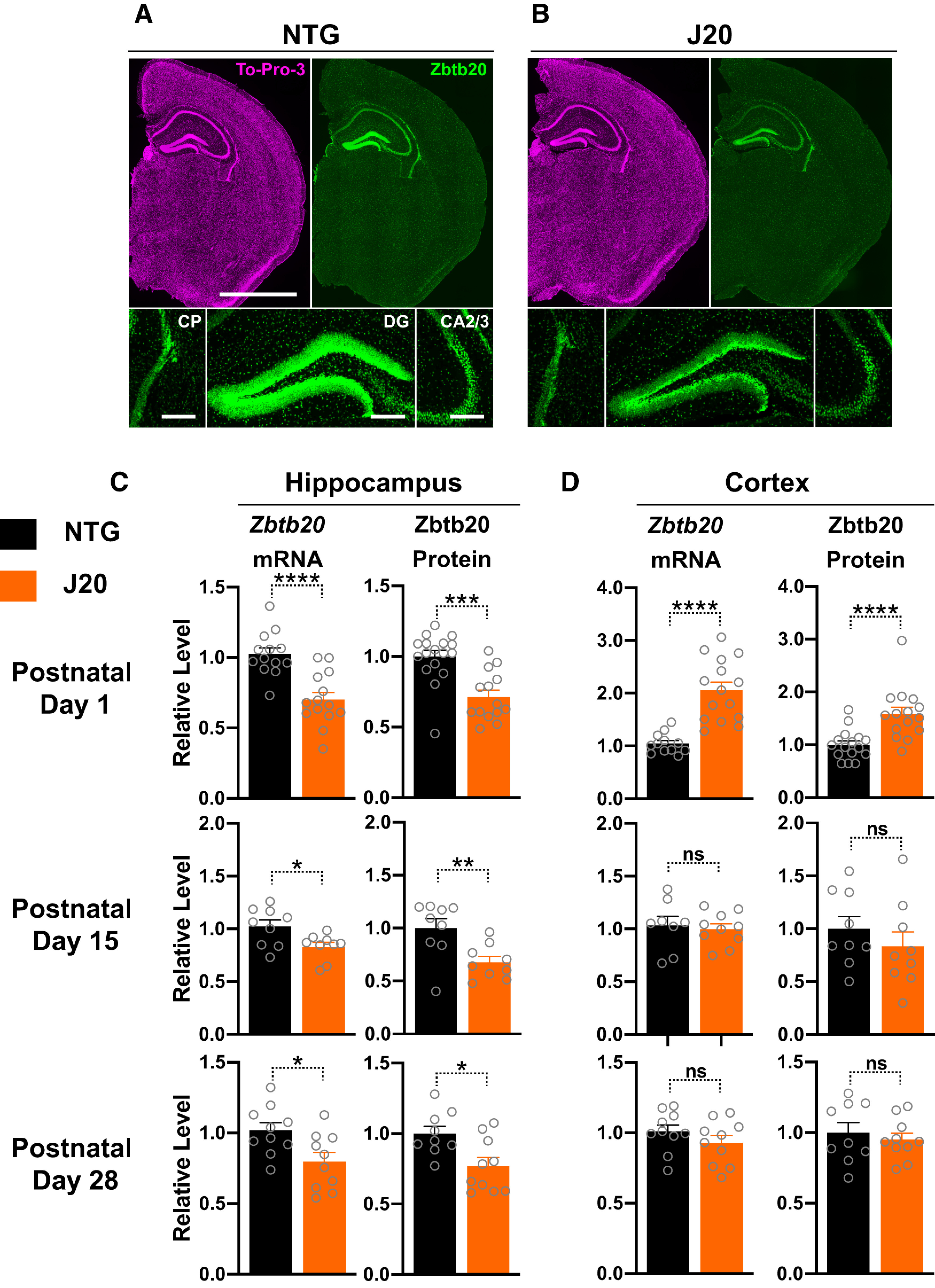
Hippocampal and cortical Zbtb20 expression during postnatal development in hAPP-J20 mice. ***A***,***B***, Coronal brain sections from three- to four-week-old mice of the indicated genotypes were stained with TO-PRO-3 (top-left) to identify nuclei, labeled with an antibody against Zbtb20 (top-right and bottom rows), and imaged by epifluorescence microscopy as in Figure 2. Hemibrain sections (scale bar: 2 mm) are shown at the top and magnified views (scale bar: 200 μm) of the indicated brain regions at the bottom. ***C***,***D***, Zbtb20 mRNA and protein levels in the hippocampus (***C***) and cortex (***D***) of hAPP-J20 mice and NTG littermate controls at postnatal days 1 (top), 15 (middle), or 28 (bottom) were measured by RT-qPCR (left) and Western blot (right) analysis, respectively, using tissues from opposite hemibrains of the same mice. Relative *Zbtb20* mRNA levels were determined as in Figure 2 except that mean *Zbtb20/Gapdh* mRNA ratios in matching brain regions of NTG mice were defined as 1.0. Relative Zbtb20 protein levels were normalized to β-tubulin levels and mean Zbtb20/β-tubulin ratios in matching brain regions of WT mice were defined as 1.0; *n* = 8–17 mice per group; *p <* *0.05, **p <* *0.01, ***p <* *0.001, ****p <* *0.0001 by two-tailed permutation test (***C***, postnatal day 1, protein), unpaired two-tailed *t* test with Welch correction (***D***, postnatal day 1, mRNA), or unpaired two-tailed Student’s *t* test (***C***,***D***, all other graphs). ns, not significant. Dots represent individual mice and bars are means ± SEM.

**Figure 5.**
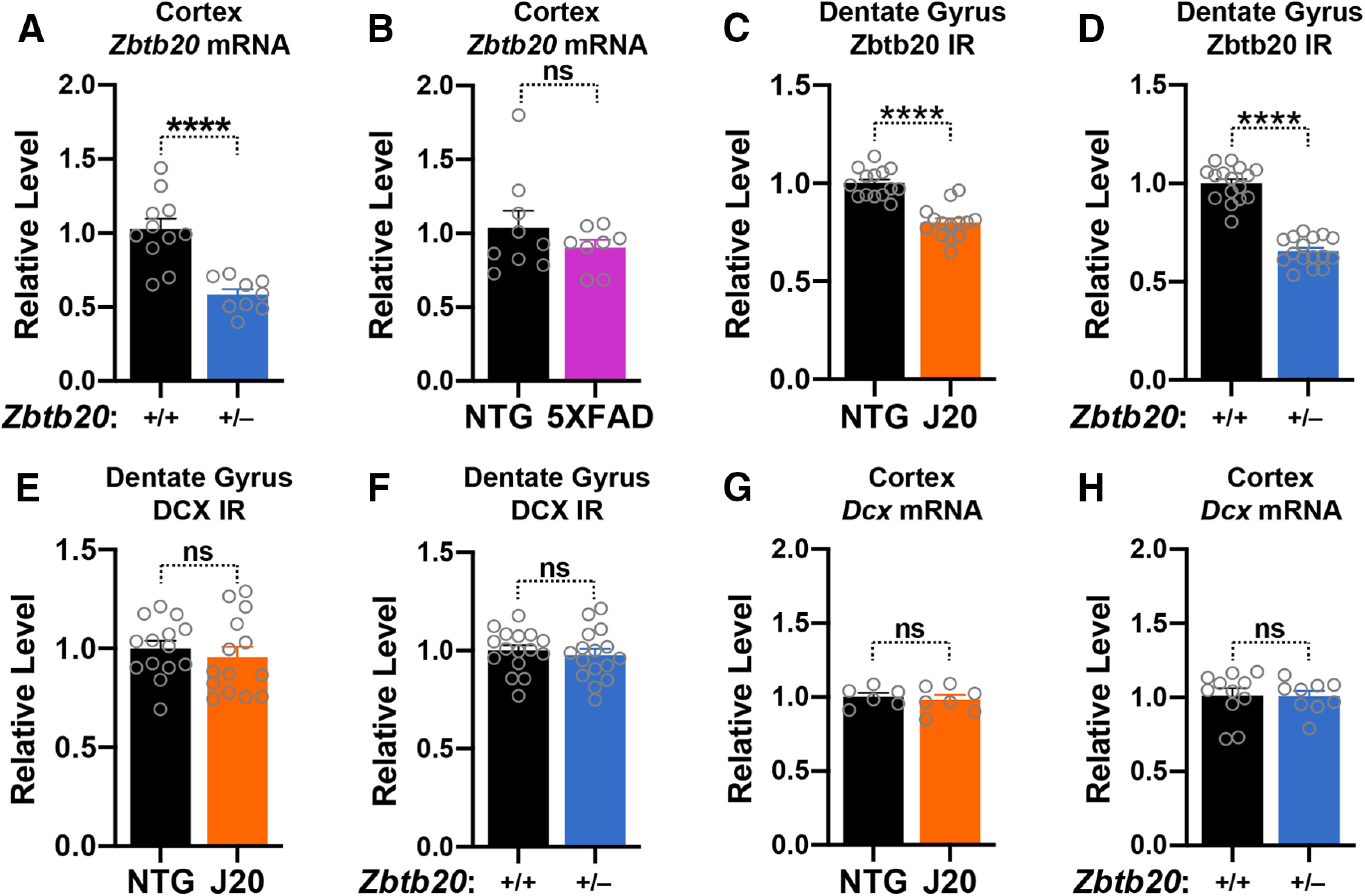
Zbtb20 and doublecortin levels in the cortex and dentate gyrus of *Zbtb20*^+/–^ mice, 5XFAD mice, hAPP-J20 mice, and controls. ***A***,***B***, Cortical levels of *Zbtb20* mRNA were measured in mice of the indicated genotypes on postnatal day 1 as described in Figure 3. ***C–F***, Coronal brain sections from three- to four-week-old mice of the indicated genotypes were co-immunostained for Zbtb20 and doublecortin (DCX) and imaged by confocal microscopy. Relative Zbtb20 (***C***,***D***) and doublecortin (***E***,***F***) immunoreactivity (IR; signal intensity) in the granular layer of the dentate gyrus. Mean levels in NTG or WT controls were defined as 1.0. ***G***,***H***, Cortical levels of doublecortin (*Dcx*) mRNA were measured in mice of the indicated genotypes on postnatal day 1. *Dcx/Gapdh* mRNA ratios in NTG or WT mice were defined as 1; *n* = 6–16 mice per group; ****p <* *0.0001 by unpaired two-tailed Student’s *t* test (***A*, *C–H***) or unpaired two-tailed *t* test with Welch correction (***B***). ns, not significant. Dots represent individual mice and bars are means ± SEM.

Because the hAPP transgene that was used to generate hAPP-J20 mice integrated into intron 2 of one *Zbtb20* allele (Fig. 1*B*; Tosh et al., 2018; Goodwin et al., 2019), we assessed Zbtb20 expression levels in brains of hAPP-J20 mice. Between 1 and 28 d of age, Zbtb20 expression in the hippocampus and choroid plexus was lower in hAPP-J20 mice than NTG controls (Fig. 4*A–C*; data not shown). In contrast to *Zbtb20*^+/–^ mice (Fig. 2*J*,*K*), cortical Zbtb20 mRNA and protein levels in hAPP-J20 mice were comparable to those of NTG controls at 15 and 28 d (Fig. 4*D*). For unclear reasons, cortical Zbtb20 mRNA and protein levels were actually higher in newborn hAPP-J20 than NTG mice (Fig. 4*D*), an alteration that was not observed in newborn *Zbtb20*^+/–^ mice or in newborn 5XFAD mice (Fig. 5*A*,*B*), another transgenic mouse model overexpressing FAD-mutant hAPP (Oakley et al., 2006).

Because Zbtb20 is strongly expressed in doublecortin-positive granule cells of WT mice (Fig. 2*D*), we quantified Zbtb20 and doublecortin levels in the granular layer of the dentate gyrus in three- to four-week-old hAPP-J20, *Zbtb20*^+/–^ and WT mice. Although granule cells expressed less Zbtb20 in hAPP-J20 and *Zbtb20*^+/–^ mice than in WT controls (Fig. 5*C*,*D*), the three groups of mice had comparable doublecortin levels (Fig. 5*E*,*F*). In a similar vein, neither the transient increase in cortical Zbtb20 expression in hAPP-J20 mice (Fig. 4*D*) nor the reduction in cortical *Zbtb20* expression in *Zbtb20*^+/–^ mice (Fig. 5*A*) altered cortical doublecortin (*Dcx*) mRNA levels in neonatal mice (Fig. 5*G*,*H*). Together, these results make it unlikely that Zbtb20 regulates neuronal doublecortin levels during early postnatal development.

**Figure 6.**
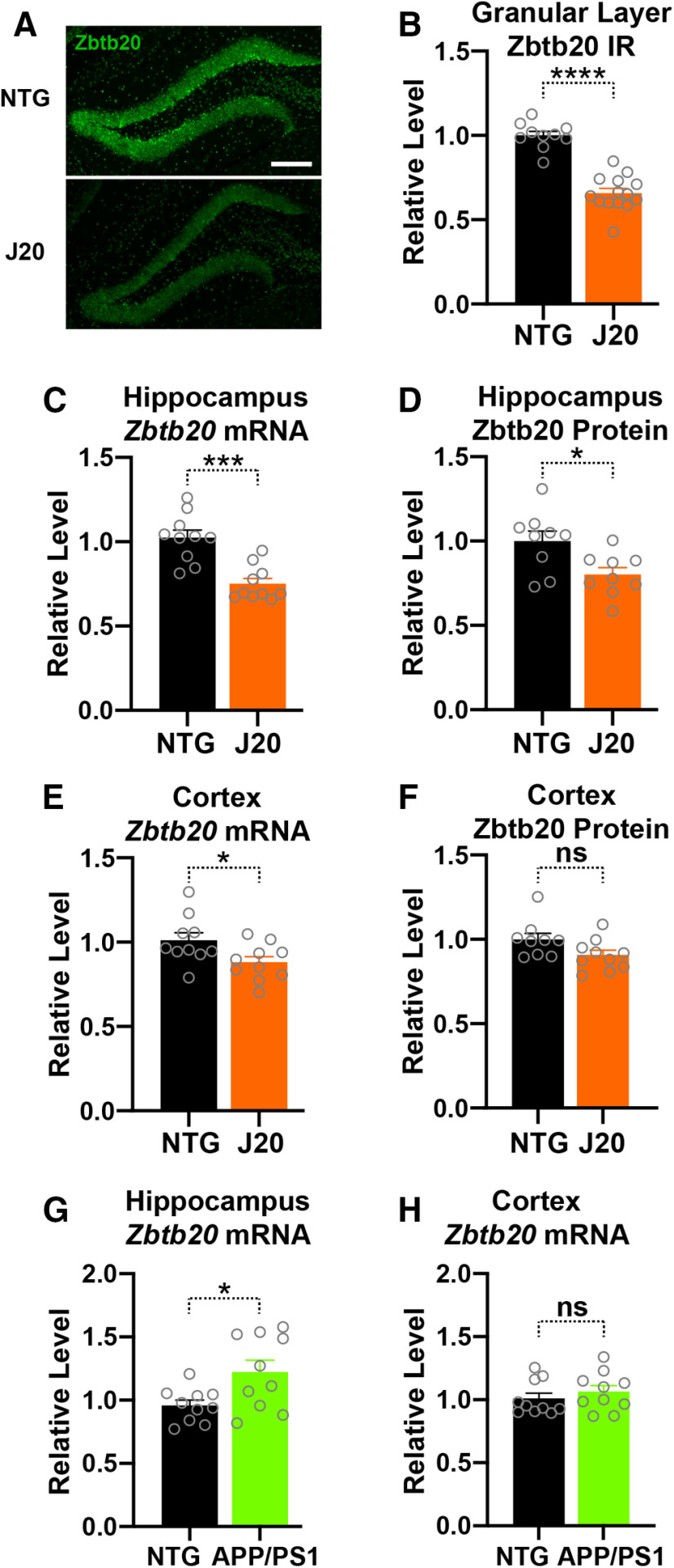
Hippocampal and cortical Zbtb20 expression in adult hAPP-J20 mice and NTG controls. ***A***, Representative confocal photomicrographs of coronal dentate gyrus sections from four- to five-month-old NTG and hAPP-J20 mice immunostained for Zbtb20 (scale bar: 200 μm). ***B***, Relative Zbtb20 immunoreactivity (IR; signal intensity) in the granular layer of the dentate gyrus in four- to five-month-old NTG and hAPP-J20 mice. Mean signals in NTG mice were defined as 1.0. ***C–F***, Zbtb20 mRNA and protein levels in the hippocampus and cortex of six-month-old NTG and hAPP-J20 mice were determined by RT-qPCR and western blot analysis, respectively, as in Figure 3. ***G***,***H***, *Zbtb20* mRNA levels in the hippocampus and cortex of six- to seven-month-old NTG and APP/PS1 mice determined by RT-qPCR; *n* = 4–6 male and 5–8 female mice per group; *p <* *0.05, ***p <* *0.001, ****p <* *0.0001 by unpaired two-tailed Student’s *t* test. ns, not significant. Dots represent individual mice and bars are means ± SEM.

At four to five months of age, Zbtb20 immunoreactivity levels in the granular layer of the dentate gyrus were still lower in hAPP-J20 mice than NTG controls (Fig. 6*A*,*B*). At six months, hippocampal levels of *Zbtb20* mRNA and Zbtb20 protein were also lower in hAPP-J20 than NTG mice (Fig. 6*C*,*D*). In the cortex of six-month-old hAPP-J20 mice, *Zbtb20* mRNA levels were only slightly reduced and a trend toward Zbtb20 protein reduction did not reach statistical significance (Fig. 6*E*,*F*). In six- to eight-month-old APP/PS1 mice, which also overexpress hAPP/Aβ (Jankowsky et al., 2004) but do not have an insertional *Zbtb20* mutation (Goodwin et al., 2019), *Zbtb20* mRNA levels were slightly increased in the hippocampus and unchanged in the cortex, as compared with age-matched NTG controls (Fig. 6*G*,*H*). These findings suggest that the reduced hippocampal *Zbtb20* expression in hAPP-J20 is caused by their insertional *Zbtb20* mutation rather than by overexpression of FAD-mutant hAPP or accumulation of Aβ.

### Zbtb20-expressing cell types

**Figure 7.**
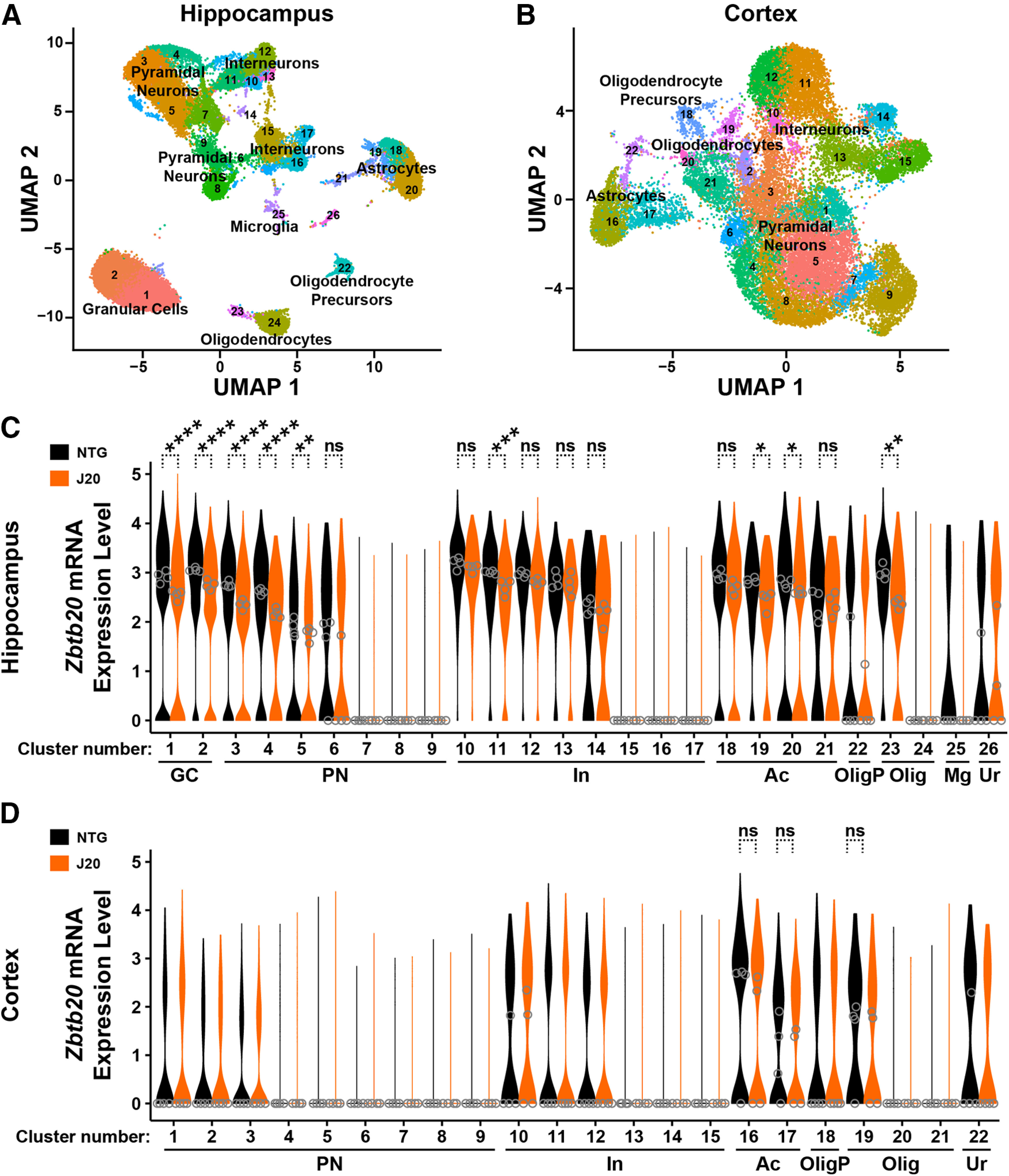
*Zbtb20* mRNA expression in single nuclei isolated from hippocampus or cortex of hAPP-J20 mice and NTG controls. Cell nuclei were isolated from the hippocampus and cortex of eight-month-old NTG and hAPP-J20 mice and analyzed by single-nucleus RNA sequencing (snRNA-seq). ***A***,***B***, Uniform manifold approximation and projection (UMAP) plot of hippocampal (***A***) and cortical (***B***) snRNA-seq data from both groups of mice. Clustering and marker gene analysis identified 17 neuronal and nine non-neuronal hippocampal clusters and 15 neuronal and seven non-neuronal cortical clusters. Individual dots correspond to nuclei and colors to clusters. The assignment of cell types to a cluster was based on 10–20 curated differentially expressed genes. ***C***,***D***, Violin plots comparing the distribution of *Zbtb20* expression in individual hippocampal (***C***) and cortical (***D***) nuclei from NTG and hAPP-J20 mice, grouped by cluster. Normalized expression levels were calculated by dividing Zbtb20 reads by total reads for any given nucleus, multiplying by 10,000, and taking the natural log of the product. Open circles represent the median *Zbtb20* expression of all nuclei in a cluster from an individual mouse. Ac; astrocytes; GC, dentate gyrus granule cells; In, interneurons; Mg, microglia; Olig, oligodendrocytes; OligP, oligodendrocyte precursors; PN, pyramidal neurons; Ur, unresolved; *n* = 4 female mice per group. The significance of genotype effects on *Zbtb20* expression was determined in clusters whose medians of *Zbtb20* expression were >0 in ≥3 WT mice, using individual mice as the number of biological samples (*n*); *p <* *0.05, **p <* *0.01, ***p <* *0.001, ****p <* *0.0001 by pseudo-bulk analysis in *muscat* (Crowell et al., 2020) with Holm–Sidak correction. ns, not significant.

To ascertain which cell types had reduced Zbtb20 expression in hAPP-J20 mice, we isolated cell nuclei from the hippocampus and cortex of hAPP-J20 and NTG mice and analyzed them by single-nucleus RNA sequencing (snRNA-seq). Clustering analysis of the hippocampal and cortical snRNA-seq data identified 26 and 22 distinct clusters, respectively, that, based on cell type-specific markers, likely represent dentate gyrus granule cells, pyramidal cells, interneurons, astrocytes, oligodendrocyte precursors, oligodendrocytes, and microglia (Fig. 7*A*,*B*). Consistent with our immunohistochemical analysis (Fig. 2*F–I*), *Zbtb20* mRNA was readily detected in hippocampal neurons, astrocytes and oligodendrocytes, whereas *Zbtb20* mRNA levels were much lower in microglial cells in NTG and hAPP-J20 mice (Fig. 7*C*). In both genotypes, *Zbtb20* mRNA expression was much lower in some neuronal and oligodendroglial clusters than others (Fig. 7*C*), highlighting the heterogeneity of cell populations in the brain. Compared with NTG controls, hAPP-J20 mice had reduced *Zbtb20* mRNA levels in some clusters of granule cells, pyramidal cells, interneurons, astrocytes, and oligodendrocytes (Fig. 7*C*). However, because the number of mice per genotype we were able to analyze with this methodology was small, these preliminary findings need to be validated in additional studies. As expected from our immunohistochemical (Figs. 2*A*, 4*A*), RT-qPCR (Fig. 2*J*), and Western blot (Fig. 2*K*) analyses, *Zbtb20* mRNA levels in most cell types were lower in cortical than hippocampal nuclei (Fig. 7*C*,*D*), and this difference was independent of genotype. Thus, if the insertional *Zbtb20* mutation were contributing to AD-relevant phenotypes in hAPP-J20 mice, it would likely manifest in hippocampus-dependent processes, especially those dependent on pyramidal neurons or dentate granule cells.

### *Zbtb20*^+/–^ mice lack the severe behavioral abnormalities observed in hAPP-J20 mice

**Figure 8.**
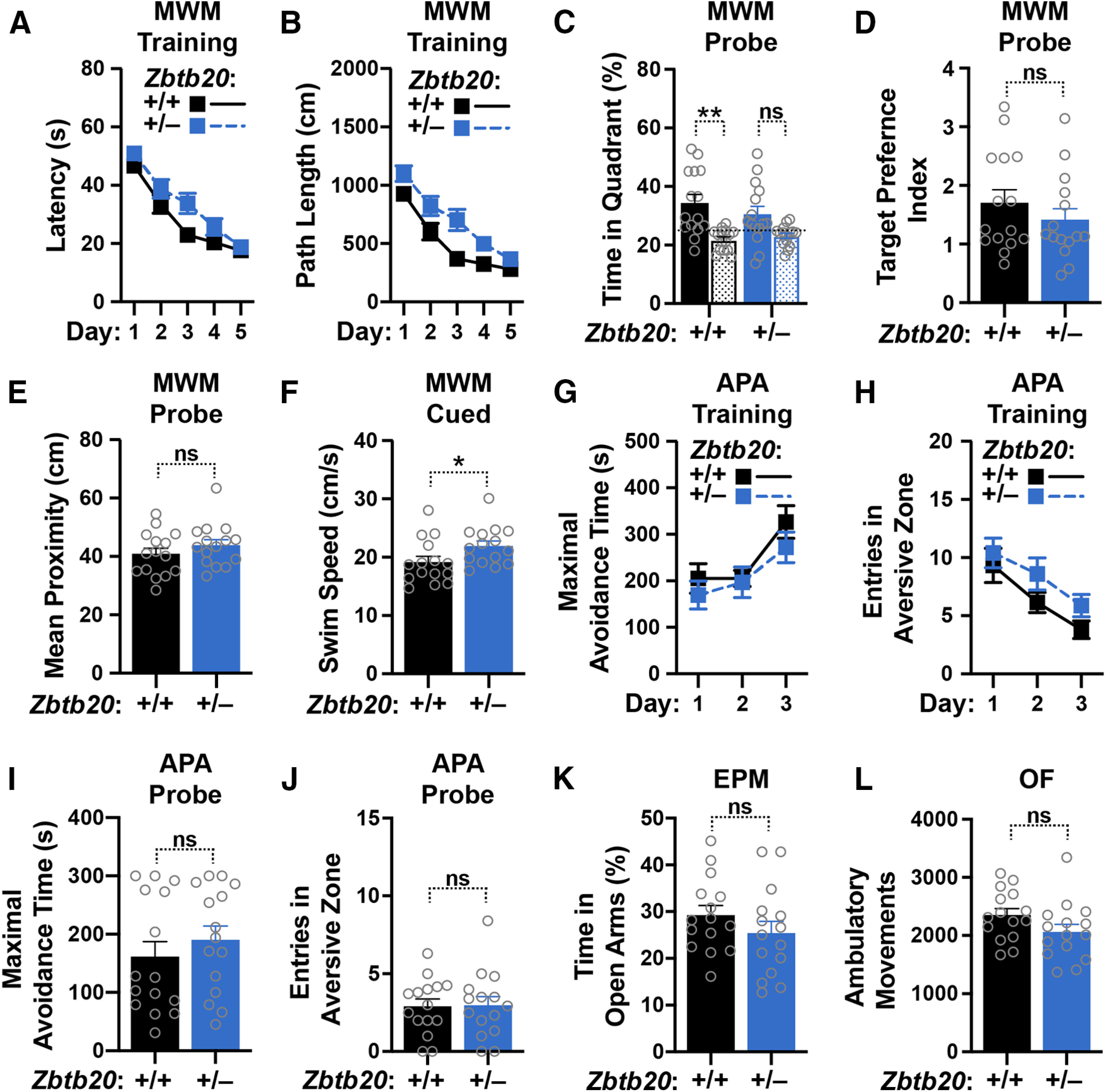
Behavioral performance of *Zbtb20*^+/–^ mice and WT controls. ***A–L***, *Zbtb20*^+/–^ mice and WT (*Zbtb20*^+/+^) controls were tested in the Morris water maze (MWM), active place avoidance (APA), elevated plus maze (EPM), and open field (OF) paradigms at five to seven months of age. ***A–F***, Learning of the spatial component (platform hidden) of the MWM task as indicated by reductions in the escape latency (***A***) and path length (***B***) to reach a hidden platform. ***C–F***, A probe trial (platform removed) was performed 24 h after the last training trial to measure the percent time mice spent in the target (solid) versus non-target (stippled) quadrants (***C***), calculate the target preference index (ratio of target/non-target percentages; ***D***), and determine the average distance of mice from the original target position during the trial (***E***). ***F***, Swim speed during the second trial in the visually cued component of the MWM. ***G–J*,** Learning of the APA task as indicated by increases in maximal avoidance time (***G***) and reduced numbers of entries into the aversive zone (***H***). ***I***,***J***, A probe trial (aversive stimulus inactivated) was performed 24 h after the last training trial to measure the maximal avoidance time of (***I***) and number of entries into (***J***) what was the aversive zone during training. ***K***, Percent time spent in the open arms of the EPM. ***L***, Ambulatory movements (beam breaks) during 15 min in the OF; *n* = 15 male mice per group. Linear mixed model analysis of learning curves revealed significant effects of training (day) in (***A***, *p *<* *0.0001; ***B***, *p *<* *0.0001; ***G***, *p *<* *0.001; ***H***, *p *<* *0.0001) and of genotype in (***B***, *p *<* *0.01) but not (***A***, *p *=* *0.1; ***G***, *p *=* *0.6; ***H***, *p *=* *0.4). No interactions were detected between training and genotype (***A***, *p *=* *0.4; ***B***, *p *=* *0.3; ***G***, *p *=* *0.6; ***H***, *p *=* *0.6). *p <* *0.05, **p <* *0.01 by paired (***C***) or unpaired (***D–F***,***I–L***) two-tailed Student’s *t* test. ns, not significant. Dots represent individual mice and bars are means ± SEM.

Although the results described above clearly suggest that hAPP-J20 mice have lower Zbtb20 expression in hippocampal neurons and other cell types, it is unclear whether these changes contribute to the development of AD-like functional abnormalities in this model. We therefore examined whether the ∼50% reduction of Zbtb20 levels in *Zbtb20*^+/–^ mice reproduces any of the behavioral alterations observed in hAPP-J20 mice. In hAPP-J20 mice, deficits in learning and memory become detectable around three to four months of age and are prominent by five to seven months (Palop et al., 2003; Esposito et al., 2006; Cheng et al., 2007; Deipolyi et al., 2008). For example, in the Morris water maze paradigm, which measures hippocampus-dependent spatial learning and memory, hAPP-J20 mice take longer and travel a greater distance to find the hidden platform during training trials; and subsequently, they spend less time searching in the target quadrant after the target platform is removed from the pool (probe trial; Palop et al., 2003; Deipolyi et al., 2008; Verret et al., 2012; Johnson et al., 2020). In this test, five- to seven-month-old *Zbtb20*^+/–^ mice showed only subtle deficits in task acquisition as compared with age-matched WT controls (Fig. 8*A*,*B*). In the probe trial, *Zbtb20*^+/–^ mice showed a trend (*p *=* *0.06) toward favoring the target quadrant slightly less than WT mice (Fig. 8*C*), but no significant differences were found between the groups during this component of the test in target preference index or mean proximity to the original platform location (Fig. 8*D*,*E*). Swim speeds were measured while mice were trained to locate a visually cued platform and were slightly higher in *Zbtb20*^+/–^ mice than in WT controls (Fig. 8*F*), which is also in contrast to hAPP-J20 mice, whose swim speeds are similar to those of controls (Palop et al., 2003; Esposito et al., 2006; Johnson et al., 2020).

Active place avoidance is another robust test of spatial learning and memory (Cimadevilla et al., 2001). In this paradigm, hAPP-J20 mice show prominent reductions in maximal avoidance time and more entries into the aversive zone than WT controls, both during training and in the probe trial when electrical shocks are no longer delivered in what was the aversive zone during training (Johnson et al., 2020). In contrast, five- to seven-month-old *Zbtb20*^+/–^ mice had no difficulties learning this task (Fig. 8*G*,*H*). *Zbtb20*^+/–^ mice also performed as well as WT controls in the probe trial (Fig. 8*I*,*J*).

In the elevated plus maze, which provides measures of anxiety and exploratory behaviors (Walf and Frye, 2007), hAPP-J20 mice avoid the open arms of the maze less than WT controls, possibly reflecting reduced anxiety, disinhibition, or deficits in avoidance learning (Chin et al., 2005; Esposito et al., 2006; Cheng et al., 2007; Roberson et al., 2007; Sanchez et al., 2012; Johnson et al., 2020). No significant differences were observed in this measure between five- to seven-month-old *Zbtb20*^+/–^ mice and WT controls (Fig. 8*K*). In the open field, which measures exploration and locomotor activity, hAPP-J20 mice are hyperactive (Cheng et al., 2007; Sanchez et al., 2012; Johnson et al., 2020), whereas *Zbtb20*^+/–^ mice are not (Fig. 8*L*).

Thus, 50% reduction of Zbtb20 expression in all brain regions and cell types does not reproduce the prominent behavioral alterations observed in hAPP-J20 mice. In contrast, expression of hAPP or specific hAPP metabolites from other sites within the genome has been shown to cause similar behavioral alterations in other lines of transgenic and knock-in mice (Hsiao et al., 1996; Holcomb et al., 1999; Chishti et al., 2001; King and Arendash, 2002; Van Dam et al., 2003; Hartman et al., 2005; Ohno et al., 2006; Ittner et al., 2010; Roberson et al., 2011; Jawhar et al., 2012; Merlini et al., 2019; Johnson et al., 2020). Together, these results suggest that Zbtb20 hypofunction does not significantly contribute to the pathogenesis of cognitive deficits and behavioral abnormalities in hAPP-J20 mice.

### No premature mortality or prominent epileptiform activity in *Zbtb20*^+/–^ mice

**Figure 9.**
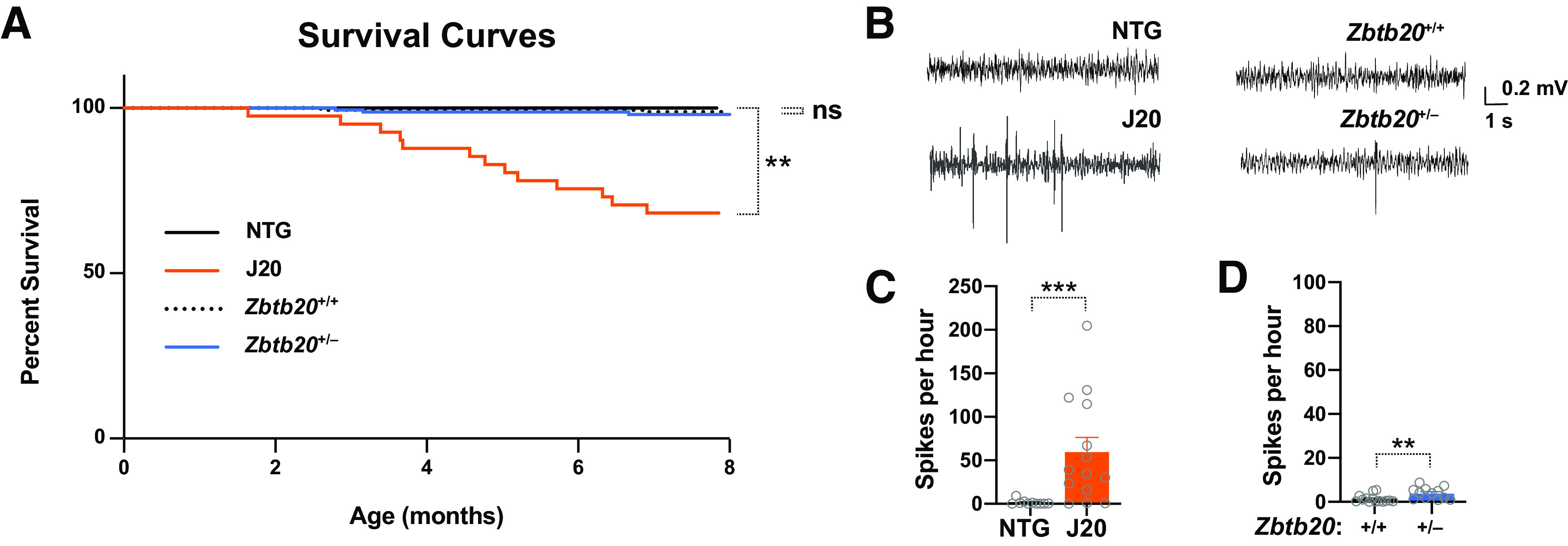
Survival and EEG activity in hAPP-J20 mice, *Zbtb20*^+/–^ mice, and controls. ***A***, Kaplan–Meier survival curves of the indicated genotypes. The hAPP-J20 and NTG data were described in a previous publication (Johnson et al., 2020). ***B–D***, Intracranial EEG recordings were obtained from resting mice of the indicated genotypes at 9–11 months of age. ***B***, Representative traces depicting multiple epileptiform spikes in an hAPP-J20 mouse (bottom-left), a single spike in a *Zbtb20*^+/–^ mouse (bottom-right), and normal EEG activity in control mice (top). ***C***,***D***, Spike frequencies measured in hAPP-J20 (***C***) and *Zbtb20*^+/–^ (***D***) mice and age-matched controls from each of these lines (***C***,***D***) while they were resting. Note the different *y*-axis scales in these panels; *n* = 12–14 male mice per group; **p <* *0.01, ***p <* *0.001 by Mantel–Cox log-rank test (***A***) or unpaired two-tailed Student’s *t* test (***C***,***D***). ns, not significant. Dots represent individual mice and bars are means ± SEM.

Between 20% and 40% of hAPP-J20 mice die prematurely between birth and six to eight months of age, most likely because of epileptic activity (Cheng et al., 2007; Roberson et al., 2007; Palop and Mucke, 2016; Johnson et al., 2020). Mice with complete genetic ablation of *Zbtb20* die by four weeks of age, most likely as a result of metabolic abnormalities (Sutherland et al., 2009).To assess whether reductions in *Zbtb20* expression might contribute to the premature mortality of hAPP-J20 mice, we monitored the survival of *Zbtb20*^+/–^ mice and WT controls and compared the results to survival curves previously published for hAPP-J20 mice and NTG controls (Johnson et al., 2020). In contrast to hAPP-J20 mice, no premature mortality was observed in *Zbtb20*^+/–^ mice between birth and eight months of age (Fig. 9*A*). We also did not notice an increased incidence of unexplained deaths among older *Zbtb20*^+/–^ mice, as compared with age-matched WT controls (data not shown). Thus, partial reduction of Zbtb20 expression does not reproduce the premature mortality observed in hAPP-J20 mice. In contrast, overexpression of hAPP or specific hAPP metabolites from other sites within the genome has been shown to cause premature mortality in other lines of transgenic mice (Hsiao et al., 1995; Chishti et al., 2001; King and Arendash, 2002; Ittner et al., 2010; Leroy et al., 2012).

Similar to a substantial proportion of AD patients (Palop and Mucke, 2016; Vossel et al., 2016; Horváth et al., 2017), hAPP-J20 mice have non-convulsive epileptiform activity (Palop et al., 2007; Sanchez et al., 2012; Verret et al., 2012; Palop and Mucke, 2016; Johnson et al., 2020). To assess whether decreases in Zbtb20 expression might contribute to the development of epileptiform activity in hAPP-J20 mice, we obtained video-EEG recordings in 9- to 11-month-old hAPP-J20 mice, *Zbtb20*^+/–^ mice, and controls, while they were resting.

As expected based on previous findings (Palop et al., 2007; Sanchez et al., 2012; Verret et al., 2012; Palop and Mucke, 2016; Johnson et al., 2020), hAPP-J20 mice had a much higher frequency of epileptiform spikes than NTG controls (Fig. 9*B*,*C*). In contrast, spike frequencies were only minimally increased in *Zbtb20*^+/–^ mice, as compared with WT controls (Fig. 9*B*,*D*). Whereas ∼70% of hAPP-J20 mice had at least 20 spikes per hour, none of the *Zbtb20*^+/–^ mice came close to this level (Fig. 9*C*,*D*). Thus, it seems unlikely that Zbtb20 reductions are responsible for the epileptiform activity in hAPP-J20 mice, particularly since this type of network dysfunction has also been observed in many other mouse models that express hAPP or specific hAPP metabolites from other sites within the genome, including multiple lines of hAPP transgenic mice (Palop and Mucke, 2016) and *App*^NL-G-F^ knock-in mice (Johnson et al., 2020).

### Zbtb20 reduction does not alter molecular indicators of epileptiform activity in the hippocampus

**Figure 10.**
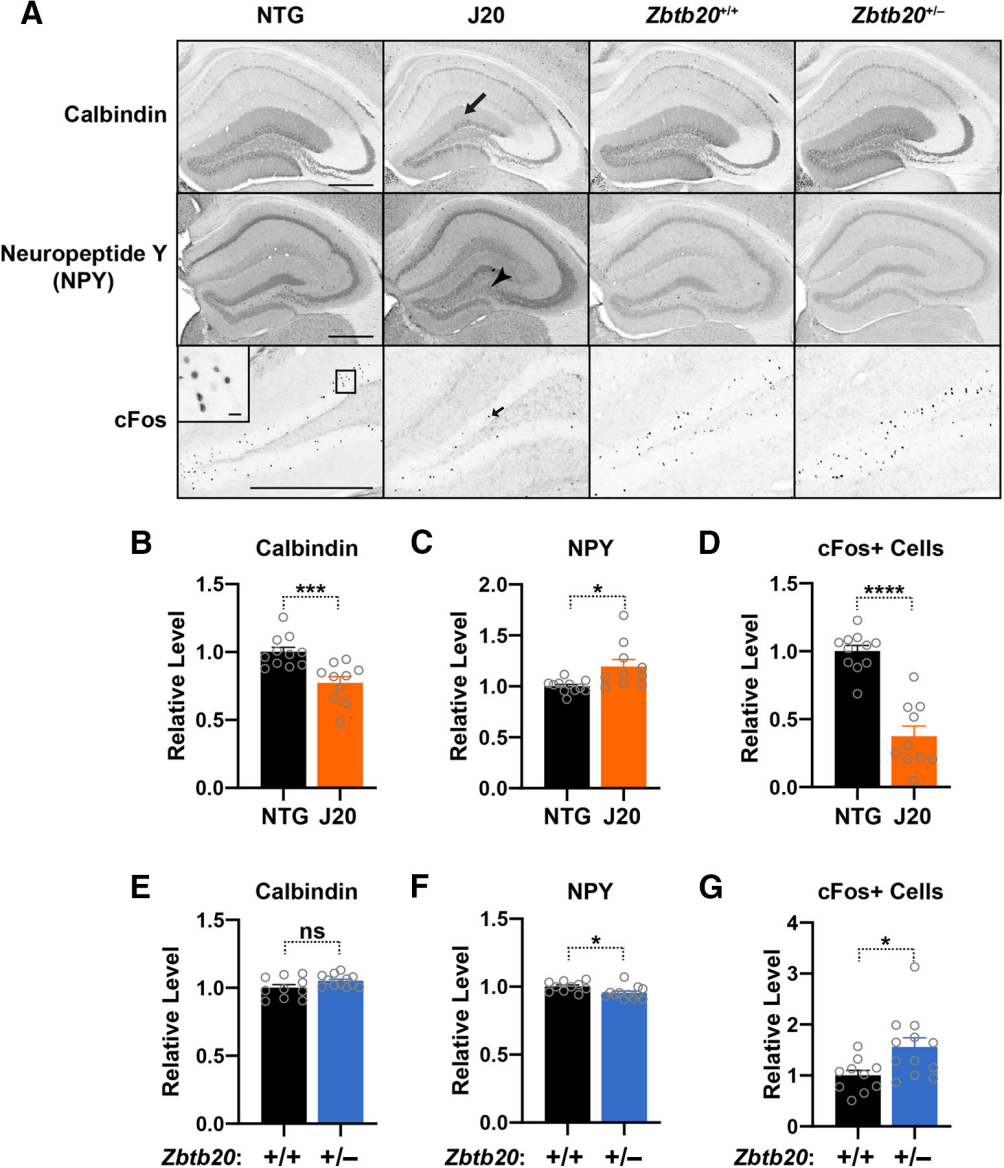
Molecular indicators of epileptiform activity. ***A–G***, Coronal brain sections from five- to eight-month-old mice of the indicated genotypes were immunostained for calbindin (***A***,***B***,***E***), NPY (***A***,***C***,***F***), or c-Fos (***A***,***D***,***G***). ***A***, Representative images depicting the levels and distributions of calbindin, NPY, and c-Fos immunoreactivities in the dentate gyrus and hippocampus. As is typical for this model, the hAPP-J20 mouse showed reduced calbindin staining in the molecular layer of the dentate gyrus (top, arrow), increased NPY in mossy fibers (middle, arrowhead), and fewer c-Fos-positive (+) cells in the granular layer (bottom, small arrow). Scale bars: 500 μm (inset, 200 μm). ***B–G***,Quantifications of these indicators of epileptiform activity. Mean levels in WT or NTG controls were defined as 1.0; *n* = 10–12 female mice per group; *p <* *0.05, ***p <* *0.001, ****p <* *0.0001 by unpaired two-tailed Student’s *t* test (***B***,***E***,***G***), unpaired two-tailed *t* test with Welch correction (***C***,***D***), or two-tailed permutation test (***F***). ns, not significant. Dots represent individual mice and bars are means ± SEM.

In models of epilepsy and in lines of mice expressing hAPP or specific hAPP metabolites, chronic epileptiform activity leads to molecular alterations in the hippocampus, including reduced expression of calbindin and c-Fos and increased expression of NPY in dentate granule cells (Palop et al., 2003, 2007, 2011; You et al., 2017; Johnson et al., 2020). In hAPP-J20 mice, these markers of epilepsy correlate well with behavioral alterations (Palop et al., 2003; Deipolyi et al., 2008; Sanchez et al., 2012). To assess whether these molecular changes in hAPP-J20 mice might be caused by reductions in hippocampal Zbtb20 levels, we analyzed brain sections from five- to eight-month-old hAPP-J20 mice, *Zbtb20*^+/–^ mice, and controls by immunohistochemistry. As expected from previous studies (Palop et al., 2003, 2007, 2011; Johnson et al., 2020), hAPP-J20 mice had reduced calbindin levels in the molecular layer of the dentate gyrus (Fig. 10*A*,*B*), increased NPY levels in mossy fibers (Fig. 10*A*,*C*), and fewer c-Fos-positive cells in the granular layer of the dentate gyrus (Fig. 10*A*,*D*), as compared with NTG controls. In contrast, *Zbtb20*^+/–^ mice had no changes in calbindin (Fig. 10*A*,*E*), decreased NPY (Fig. 10*A*,*F*), and increased numbers of c-Fos-positive cells (Fig. 10*A*,*G*), demonstrating that reduced Zbtb20 expression *per se* causes distinct changes and indicating that the molecular hippocampal alterations of hAPP-J20 mice are caused by FAD-mutant hAPP or some of its metabolites, most likely through the induction of hypersynchronous network activity (Palop et al., 2007, 2011; Palop and Mucke, 2016).

### Normal liver function in hAPP-J20 and *Zbtb20*^+/–^ mice

**Figure 11.**
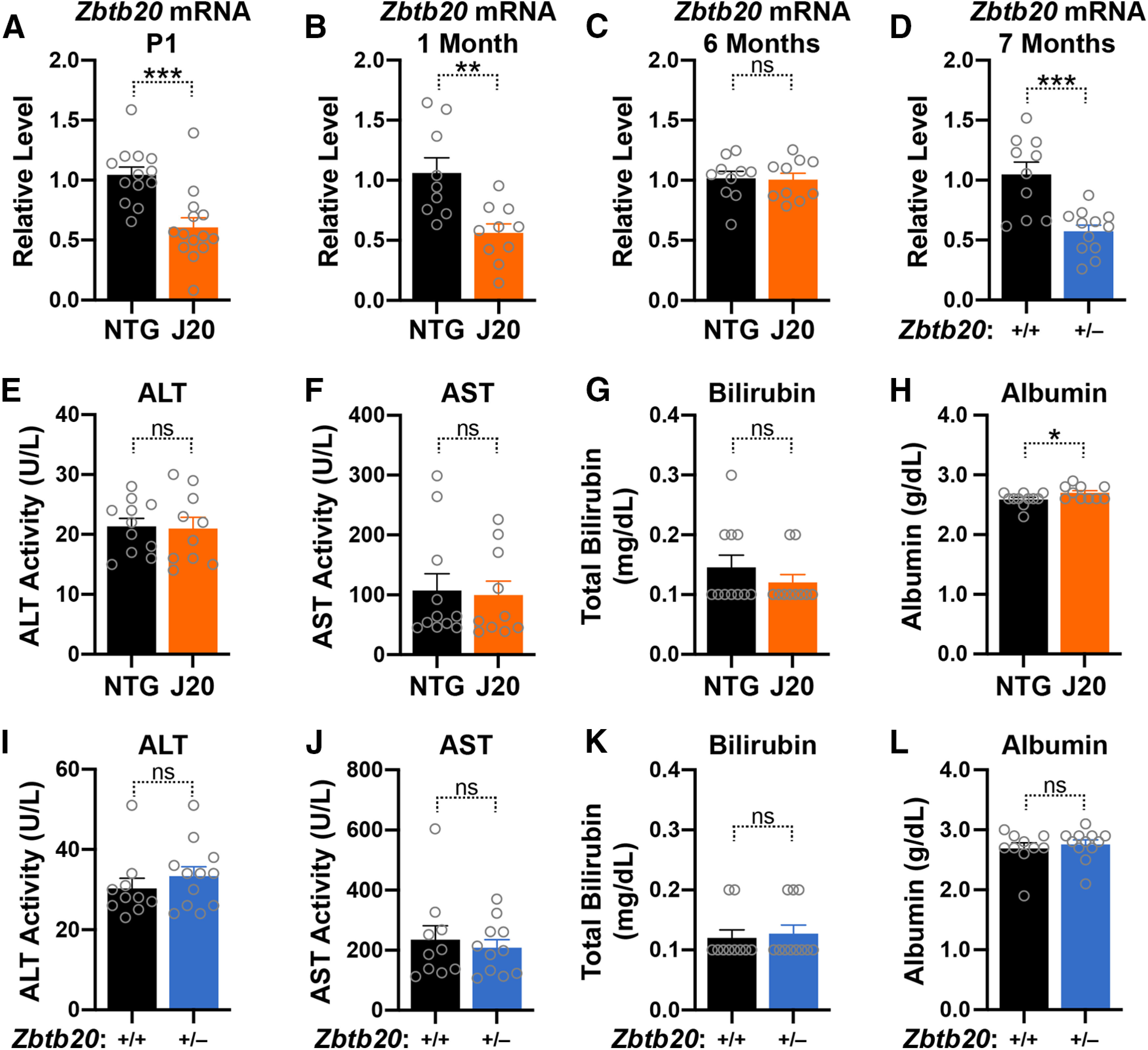
Liver levels of *Zbtb20* mRNA and serum levels of liver function indicators in hAPP-J20 mice, *Zbtb20*^+/–^ mice, and controls. ***A–D***, *Zbtb20* mRNA levels were determined in livers from NTG and hAPP-J20 mice at birth (P1; ***A***), one month (***B***), or six months (***C***) of age, and in seven-month-old *Zbtb20*^+/+^ and *Zbtb20*^+/–^ mice (***D***) by RT-qPCR using *Gapdh* mRNA as the reference. Mean levels in NTG or WT mice were defined as 1.0. (**E–L**) Serum levels of the following liver function indicators were determined in NTG and hAPP-J20 mice (***E–H***) and in *Zbtb20*^+/+^ and *Zbtb20*^+/–^ mice (***I–L***) at five to seven months of age: alanine aminotransferase (ALT) activity (***E*, *I***), aspartate transaminase (AST) activity (***F***,***J***), total bilirubin (***G***,***K***), and albumin (***H***,***L***); *p <* *0.05, **p <* *0.01, ***p <* *0.001 by unpaired two-tailed Student’s *t* test (***A–F***,***J***) or two-tailed permutation test (***G–I***,***K***,***L***). ns, not significant. Dots represent individual mice and bars are means ± SEM.

Zbtb20 is strongly expressed not only in the hippocampus, but also in the liver, which degrades many toxic metabolites. Both acute and chronic liver failure can lead to hepatic encephalopathy, a condition characterized by impaired brain functions (Felipo, 2013). In the liver, Zbtb20 functions as a transcriptional repressor and a transcriptional activator and is required for *de novo* lipogenesis, a process that converts carbohydrates into triglycerides for energy storage (Xie et al., 2008; Liu et al., 2017). To determine whether reductions of liver Zbtb20 levels might contribute to behavioral alterations in hAPP-J20 mice, we assessed liver *Zbtb20* mRNA levels in hAPP-J20 mice and NTG controls. Although liver *Zbtb20* mRNA levels in hAPP-J20 mice were reduced at birth and one month of age, they were normal at six months (Fig. 11*A–C*), when these mice show robust behavioral deficits (Palop et al., 2003; Esposito et al., 2006; Cheng et al., 2007; Roberson et al., 2007; Deipolyi et al., 2008; Sanchez et al., 2012; Verret et al., 2012; Johnson et al., 2020). In contrast, liver *Zbtb20* mRNA levels were reduced by ∼ 50% in *Zbtb20*^+/–^ mice around this age (Fig. 11*D*), providing further evidence that the insertional mutation of one *Zbtb20* allele in hAPP-J20 mice does not result in a complete knock-out of this allele. Neither hAPP-J20 mice nor *Zbtb20*^+/–^ mice had impairments in widely used indicators of liver function (Newsome et al., 2018) at five to seven months of age (Fig. 11*E–L*), indicating that behavioral alterations in hAPP-J20 mice are not because of hepatic encephalopathy.

## Discussion

These findings suggest that the insertional mutation of one *Zbtb20* allele in heterozygous hAPP-J20 mice alters *Zbtb20* expression in some brain regions, but not others, and that it does not reduce Zbtb20 expression to the same extent as ablating most of the coding sequence from one *Zbtb20* allele in *Zbtb20*^+/–^ mice. Although the insertional mutation in hAPP-J20 mice does not seem to ablate expression of the affected *Zbtb20* allele, we identified subtle effects on the complex expression of this gene in some brain regions and cell types. It is conceivable that *Zbtb20* expression in distinct brain regions is regulated differently and impacted differentially by the insertional mutation. Alternatively, the lower baseline levels of *Zbtb20* expression in the cortex may make it easier for the intact *Zbtb20* allele to compensate for the hypofunction of the mutant allele. The increased hippocampal *Zbtb20* mRNA levels we found in APP/PS1 mice raise the possibility that FAD-mutant hAPP also promotes *Zbtb20* expression in hAPP-J20 mice, an effect that could diminish the impact of the insertional mutation.

Most importantly from an AD modeling perspective, our results make it very unlikely that the profound deficits in learning and memory, behavioral abnormalities, neural network dysfunction, and related molecular alterations in hippocampal neurons of hAPP-J20 mice are caused by reduced Zbtb20 levels rather than by the expression of FAD-mutant hAPP, providing novel mechanistic insights into the pathogenesis of this model’s phenotype. Indeed, although Zbtb20 levels were more markedly reduced in *Zbtb20*^+/–^ mice than hAPP-J20 mice, *Zbtb20*^+/–^ mice had fewer and much milder behavioral alterations and distinctly less neural network dysfunction than hAPP-J20 mice, as compared with WT controls.

However, the identification of any such abnormalities in *Zbtb20*^+/–^ mice raises the possibility that even smaller extents of Zbtb20 reduction might somehow sensitize the brain to pathogenic effects of FAD-mutant hAPP and, thereby, indirectly promote the development of functional abnormalities in hAPP-J20 mice. It is worth noting in this context that mutations in *Zbtb20* cause Primrose syndrome in humans, which can be associated with intellectual impairments and seizures (Cordeddu et al., 2014; Melis et al., 2020). Excluding the possibility that reductions in Zbtb20 make minor contributions to functional alterations in hAPP-J20 mice would require complete normalization of Zbtb20 levels in hAPP-J20 mice without causing inadvertent overexpression of Zbtb20, which is difficult to achieve. Notwithstanding this caveat, we consider it likely that most alterations identified in hAPP-J20 mice result from, and provide insights into, the pathobiological activities of FAD-mutant hAPP, particularly since similar alterations have been observed in independent mouse models expressing hAPP or some of its metabolites from other sites within the genome (Hsiao et al., 1995; Hsiao et al., 1996; Holcomb et al., 1999; Chishti et al., 2001; King and Arendash, 2002; Van Dam et al., 2003; Hartman et al., 2005; Ohno et al., 2006; Ittner et al., 2010; Roberson et al., 2011; Jawhar et al., 2012; Leroy et al., 2012; Merlini et al., 2019), including in FAD-mutant *App* knock-in mice that do not overexpress hAPP (Saito et al., 2014; Orr et al., 2018; Johnson et al., 2020).

Studies to carefully differentiate between insertional effects and AD-relevant phenotypes have also been conducted in a tau transgenic model in which transgene insertions resulted in the deletion of several genes, including genes expressed in the brain (Gamache et al., 2019). It is worth noting that insertional mutations have been excluded in several (Goodwin et al., 2019) but not all of the transgenic models that are widely used in AD research. The experiments described in the current study highlight the importance of exploring inadvertent consequences of genetic modifications. Notably, it is unlikely that such consequences are restricted to transgenic models; the generation of knock-out and knock-in models could also result in unexpected genomic changes, for example, through alterations of enhancer elements acting on distant genes or of noncoding RNAs.

After the exclusion of such possible confounds, which of the available models is “best” depends on the specific question one wants to answer. Models with individual genetic modifications that were designed to study a specific factor in isolation can provide useful insights into the relative pathogenic impact of that factor, the underlying mechanisms, and potential ways to block them. However, these models may not be able to reliably predict the efficacy of therapeutic interventions in the human condition, in which multiple pathogenic factors act in concert. Negative preclinical drug trials that fail to reduce brain dysfunctions in such AD-relevant models should be taken seriously, however, because drugs that fail in a reductionist system would seem unlikely to succeed in a highly heterogeneous AD population, particularly since many neurotropic drugs with well-established efficacy in other human disorders back-translate rather well into related mouse models (Howe et al., 2018). Notably, even early treatment of hAPP-J20 mice with a BACE1 inhibitor did not prevent their network dysfunction or cognitive decline, although this intervention prevented the formation of amyloid plaques, as well as plaque associated microgliosis, and markedly reduced Aβ oligomer levels in brain tissues (Johnson et al., 2020). The current study strongly supports the conclusion that this outcome did not result from Zbtb20 deficiency causing the functional deficits of hAPP-J20 mice, an alternative mechanism that would not be expected to respond to BACE1 inhibition. In further support of this interpretation, BACE1 inhibitor treatments also failed to prevent cognitive decline in humans with AD (Egan et al., 2019; Cummings et al., 2020).

Taken together, our results suggest that Zbtb20 deficiency is not the primary driver of brain dysfunctions in hAPP-J20 mice. Because we cannot exclude the possibility that even modest degrees of Zbtb20 deficiency in some cell types sensitize mice to alterations caused by FAD-mutant hAPP or Aβ, it is advisable to validate discoveries made in the hAPP-J20 line in independent experimental models, human AD or both, as done in several previous studies (Mucke et al., 2000; Palop et al., 2003; Chin et al., 2005; Cheng et al., 2007; Roberson et al., 2011; Verret et al., 2012; Orr et al., 2015, 2018; Vossel et al., 2016; Merlini et al., 2019; Johnson et al., 2020). Validating results across independent models is prudent in general, as this strategy enhances scientific rigor as well as the likelihood that experimental findings are relevant to the human condition under study.

## Synthesis

Reviewing Editor: Karl Herrup, University of Pittsburgh School of Medicine Decisions are customarily a result of the Reviewing Editor and the peer reviewers coming together and discussing their recommendations until a consensus is reached. When revisions are invited, a fact-based synthesis statement explaining their decision and outlining what is needed to prepare a revision will be listed below. The following reviewer(s) agreed to reveal their identity: Jie Zhang.

In this manuscript, the authors compare molecular, physiological and behavioral differences among hAPP-J20 mice, Zbtb20 transgenic mice and WT mice. Their results indicate that, despite it being the site of the APP transgene insertion, Zbtb20 deficiency is not the major factor that leads to the observed brain dysfunctions in hAPP-J20 mice. Overall, the authors provide interesting data and further clarify the characteristics of this AD-like mouse. These strengths are counterbalanced to some extent by weakness which, while somewhat minor, detract from the overall impact of the work.

On page 12 (referencing Figure 2C), the authors indicate that “As shown previously, Zbtb20-/- mice had an abnormal hippocampal cytoarchitecture that was not present in Zbtb20+/- mice”. While the mRNA (Figure 2J) and western blot (Figure 2K) results are informative, given the proliferation of bands seen on the western, it would be useful if the authors could quantify the immunostaining of Zbtb20. Also, to what extent might the large number of bands seen represent post-translational modifications? Treatment of lystates with phosphatases or glycosylases before running the gel could rule out some issues. Ubiquitin staining would be another possibility.

Based on Figure 4D and Figure 7, Zbtb20 mRNA level is much higher in hAPP-J20 than NTG controls at 3-4 weeks old, however, Zbtb20 mRNA level is lower in hAPP-J20 than NTG controls at 8 months old. It would be best if the authors could discuss this discrepancy in a bit more detail and offer potential explanations.

On page 17 and in Figure 10, the authors state that “Zbtb20 reduction does not alter molecular indicators of epileptiform activity in the hippocampus”. The author compared the immunostaining result of calbindin, NPY and c-Fos-positive cells among hAPP-J20 mice, Zbtb20+/- mice and control mice. While cFos is a widely used activity marker, for the more general audience of eNeuro, the authors might want to discuss the causal links between the other two markers (NPY and calbindin).

J20 is one of several mouse models for AD that have been created. In this manuscript the authors have made the commendable effort of thoroughly investigating the importance of the site of transgene (hAPP) insertion as a contributing/complicating factor in the final mouse phenotype. The authors are understandably polite in their discussion of the lack of comparable information in most other Alzheimer's disease mouse models, but this part of the discussion could be expanded without being rude and would serve to highlight the significance of the current work. This manuscript also highlights the need to consider metabolites in establishing the final mouse phenotype. This dimension is also frequently ignored in the transgenic literature and expanding this part of the discussion a bit would guide the readers as they consider the results of other studies. In this regard, it is a bit unfortunate that the authors did not do more to explore the contribution of the choroid plexus to the phenotype.

Finally, while the authors mention the possibility in the Discussion, they never experimentally test whether haploinsufficiency of Zbtb20 (in the heterozygotes) would potentiate the pathology of other AD transgenes. This is not viewed as a requirement, but if any data were available, it would be a welcome addition to the work.

Minor point: The references to the figures should come in order. Either move Figure 7 forward (to become Figure 3) or remove the snRNA-seq reference on p.14.
