# Supplementary material for: Phenotypic Differences between the Alzheimer’s Disease-Related hAPP-J20 Model and Heterozygous Zbtb20 Knock-Out Mice
Source: eNeuro. 2021 May 10;8(3):ENEURO.0089-21.2021. doi: 10.1523/ENEURO.0089-21.2021 (PMC8121260; doi:10.1523/ENEURO.0089-21.2021)
Supplement: Extended Data Figure 1-1 — Key reagents. Download Figure 1-1, DOCX file. [file enu-eN-NWR-0089-21-s01.docx]

**Figure 1–1. Key reagents**

| **Primary Antibodies** | | | | | | | | |
| --- | --- | --- | --- | --- | --- | --- | --- | --- |
| **Antigen** | **Research**  **Resource Identifiers** | **Host Species** | **Source** | | **Catalog No.** | | **Application** | **Dilution** |
| Calbindin | AB_10000340 | Rabbit | Swant | | CB38 | | IHC | 1:30,000 |
| c-Fos [2H2] | AB_2747772 | Mouse IgG1 | Abcam | | ab208942 | | IHC | 1:2,000 |
| Doublecortin | AB_1586992 | Guinea pig | Millipore | | AB2253 | | IHC | 1:500 |
| GAPDH | AB_2107445 | Mouse | Millipore | | MAB374 | | WB | 1:10,000 |
| GFAP | AB_11212597 | Mouse | Millipore | | MAB360 | | IHC | 1:1000 |
| Iba1 | AB_839504 | Rabbit | Wako | | 019-19741 | | IHC | 1:2000 |
| NeuN | AB_2619988 | Guinea pig | Synaptic Systems | | 266 004 | | IHC | 1:1000 |
| Neuropeptide Y (NPY) | AB_2307354 | Rabbit | ImmunoStar | | 22940 | | IHC | 1:2,000 |
| Oligo2 | AB_1914109 | Rabbit | Novus Biologicals | | NBP1-28667SS | | IHC | 1:500 |
| βIII-Tubulin | AB_444319 | Rabbit | Abcam | | Ab18207 | | WB | 1:3,000 |
| β-Tubulin | AB_1850029 | Rabbit | LI-COR | | 926-42211 | | WB | 1:2,500 |
| Zbtb20 | AB_2739244 | Rat | BD Biosciences | | 565453 | | WB | 1:1,000 |
| Zbtb20 | unknown | Rabbit | Proteintech | | 23987-1-AP | | Not specific in WB | 1:1,000 |
| Zbtb20 | AB_10667543 | Rabbit | Sigma-Aldrich | | SAB2103971 | | Not specific in WB | 1:1,000 |
| Zbtb20 | AB_2808683 | Rabbit | Invitrogen | | PA5-96881 | | Not specific in WB | 1:1,000 |
| **Secondary Antibodies** | | | | | | | | |
| **Antigen** | **Conjugated Tag** | **Host Species** | **Source** | | **Catalog No.** | | **Application** | **Dilution** |
| Guinea Pig IgG | Alexa Fluor 647 | Goat | Thermo Fisher Scientific | | A21450 | | IHC | 1:500 |
| Mouse IgG | Alexa Fluor 647 | Goat | Thermo Fisher Scientific | | A-21235 | | IHC | 1:500 |
| Mouse IgG | Biotin-SP | Goat | Jackson ImmunoResearch | | 115-067-003 | | IHC | 1:500 |
| Mouse IgG | IRDye 680LT | Goat | LI-COR | | 925-68020 | | WB | 1:10,000 |
| Rabbit IgG | Biotin-SP | Donkey | Jackson ImmunoResearch | | 711-065-152 | | IHC | 1:1,000 |
| Rabbit IgG | HRP | Goat | Thermo Fisher Scientific | | G21234 | | IHC | 1:500 |
| Rabbit IgG | IRDye 680LT | Goat | LI-COR | | 925-68021 | | WB | 1:10,000 |
| Rabbit IgG | IRDye 800CW | Donkey | LI-COR | | 926-32213 | | WB | 1:10,000 |
| Rat IgG | IRDye 800CW | Goat | LI-COR | | 925-32219 | | WB | 1:10,000 |
| Rat IgG | Alexa Fluor 488 | Goat | Thermo Fisher Scientific | | A-11006 | | IHC | 1:500 |
| **Counter Stains for Immunofluorescence** | | | | | | | | |
| **Stain** | **Source** | | **Catalog No.** | | | | **Application** | **Dilution** |
| TO-PRO-3 | Thermo Fisher Scientific | | T3605 | | | | IHC | 1:10,000 |
| **Primers** | | | | | | | | |
| **Name** | | **Sequence** | | | | | | |
| Zbtb20 total forward | | 5’ – GCCCTCATCCACTCGACACA – 3’ | | | | | | |
| Zbtb20 total reverse | | 5’ – GTGAATGCGCTCGGTCATCC –3’ | | | | | | |
| Gapdh total forward | | 5’ – GGGAAGCCCATCACCATCTT – 3’ | | | | | | |
| Gapdh total reverse | | 5’ – GCCTTCTCCATGGTGGTGAA – 3’ | | | | | | |
| **TaqMan Probes** | | | | | | | | |
| **Name** | | **Source** | | **Catalog No.** | | **Assay ID** | | |
| Zbtb20 total – FAM | | Thermo Fisher Scientific | | 4331182 | | Mm00457764 | | |
| Doublecortin – FAM | | Thermo Fisher Scientific | | 4331182 | | Mm00438400 | | |
| Gapdh total – VIC | | Thermo Fisher Scientific | | 4448484 | | Mm99999915 | | |
